# Supplementary figures and images for: Digital adherence technology for tuberculosis treatment supervision: A stepped-wedge cluster-randomized trial in Uganda
Source: PLoS Med. 2021 May 6;18(5):e1003628. doi: 10.1371/journal.pmed.1003628 (PMC8136841; doi:10.1371/journal.pmed.1003628)

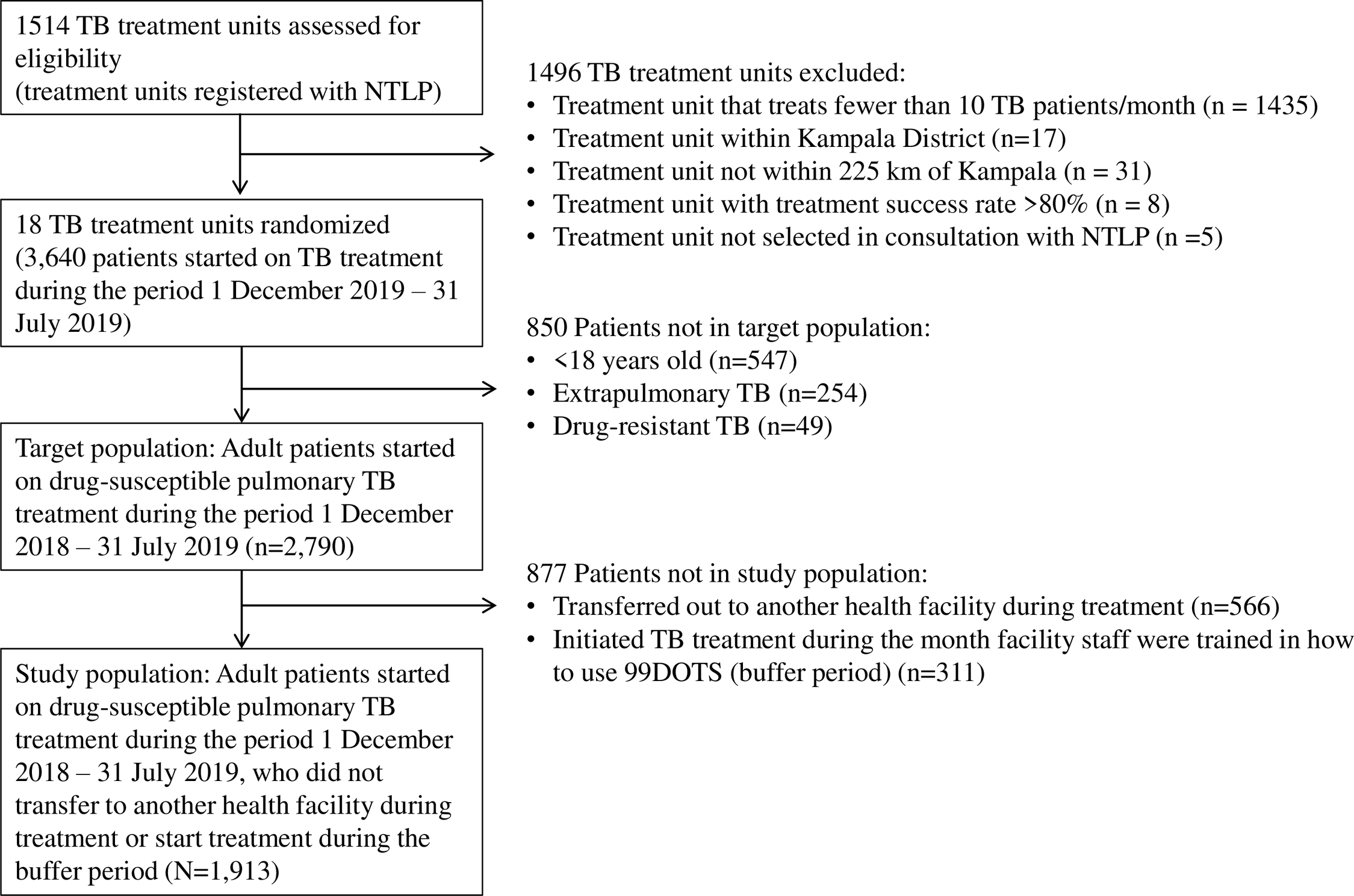

Supplement: S1 Fig — (TIF) [file pmed.1003628.s003.tif]

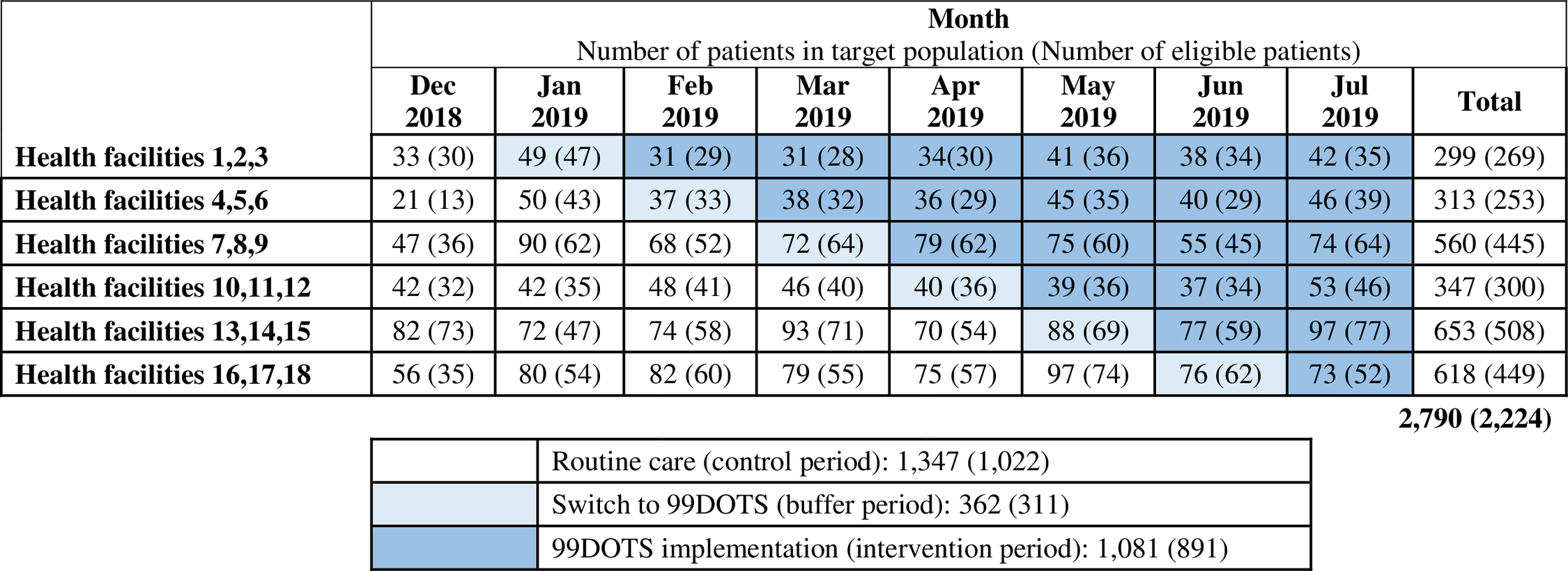

Supplement: S2 Fig — The target population includes all adults initiating treatment for drug-susceptible pulmonary TB. The eligible population excludes patients in the target population who were transferred out to another health facility during their treatment. Patients who initiated treatment during the buffer period were excluded from the study population. (TIF) [file pmed.1003628.s004.tif]

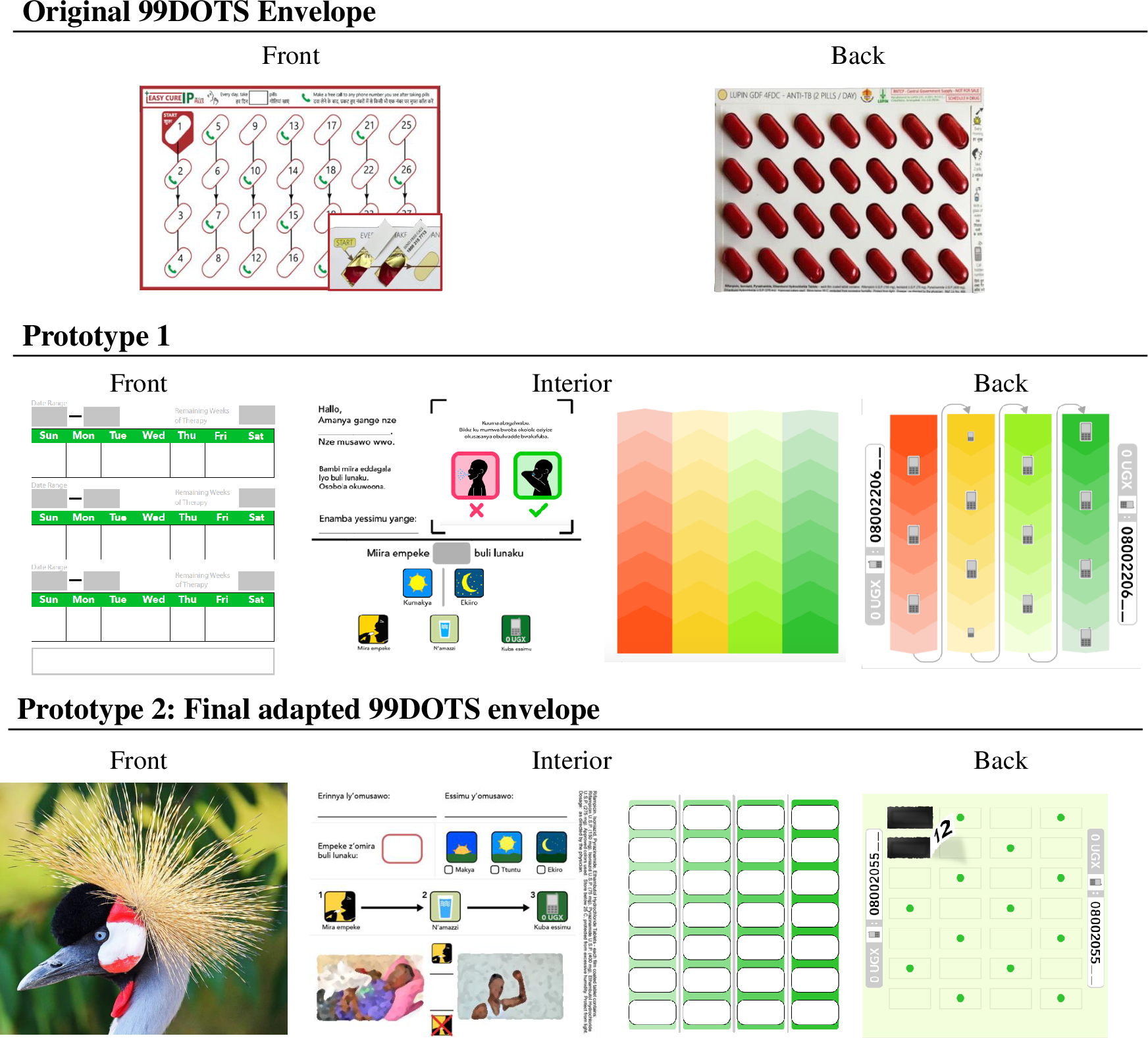

Supplement: S3 Fig — The original 99DOTS envelope (top) had 2 sides. We redesigned the original envelope using human-centered design to reduce stigma, encourage appropriate dosing, and facilitate communication between patients and health workers. Prototype 1 (middle) was used from January to June 2019. Prototype 2 (bottom) was used from July 2019 through the end of the trial. In addition to the changes shown here, the ring tone heard when patients called toll-free numbers to self-report dosing was replaced with a rotating series of educational or motivational messages recorded by local health workers. (TIF) [file pmed.1003628.s005.tif]
