# Supplementary material for: Digital adherence technology for tuberculosis treatment supervision: A stepped-wedge cluster-randomized trial in Uganda
Source: PLoS Med. 2021 May 6;18(5):e1003628. doi: 10.1371/journal.pmed.1003628 (PMC8136841; doi:10.1371/journal.pmed.1003628)
Supplement: S1 Statistical Analysis Plan — (DOCX) [file pmed.1003628.s006.docx]

**Statistical Analysis Plan: DOT to DAT**

| **Full Title** | From Directly-Observed Therapy (DOT) to Digital Adherence Technology (DAT) for TB Treatment: The DOT to DAT Trial | | |
| --- | --- | --- | --- |
| **Acronym** | DOT to DAT | | |
| **Document History** | **Version No.** | **Version Date** | **Description of Change** |
|  | 1.0 | 2019-11-05 | Initial release |
|  | 1.1 | 2020-02-06 | Clarifying dataset assembly, removed conversion from secondary outcomes (not reliably available in treatment register) |
|  |  |  |  |
|  |  |  |  |
|  |  |  |  |
| **Trial Registration** | Pan African Clinical Trials Registry (PACTR201808609844917) | | |
| **Principal Investigators** | Adithya Cattamanchi, Achilles Katamba | | |

# Introduction

## Aim

To determine whether a 99DOTS-based strategy improves TB treatment outcomes compared to routine care in Uganda.

# Background and Objectives

## Specific Objectives

1. Determine whether a 99DOTS-based strategy improves TB treatment outcomes.
   1. *Strategy:*
      1. Confirmation of dosing via toll-free phone calls using 99DOTS
      2. SMS dosing reminders to patients delivered via 99DOTS
      3. Weekly two-way check-in via interactive voice response using 99DOTS
      4. Differential management based on dosing history and response to weekly check-in.
   2. *Standard-of-care*: routine community- or facility-based treatment via directly-observed therapy (DOT)
2. Evaluate reach, adoption and implementation of the 99DOTS-based strategy.
3. Evaluate the incremental costs, outcomes, and cost-effectiveness associated with 99DOTS as compared to the standard of care from the health system and patient perspectives.

## Summary of aims

## General comments

This statistical analysis plan covers the primary effectiveness and key secondary effectiveness and implementation outcomes. These endpoints will be evaluated through review of TB treatment registers and TB treatment cards for all patients initiating TB treatment at participating health facilities, and through review of the 99DOTS server. Trial results reporting will follow the CONSORT 2010 statement extension for stepped wedge trials.^[1](#_ENREF_3)^ This Statistical Analysis Plan is written in support of and is predominantly consistent with the full trial protocol; however, this analysis plan takes precedence.

# Trial Summary

## Trial design

The study proposes to conduct a pragmatic, stepped-wedge randomized trial to evaluate the effectiveness and implementation of a 99DOTS-based strategy relative to standard supervision of TB treatment. The effectiveness of the 99DOTS-based strategy will be assessed using routine data collected as part of mandatory reporting to the Uganda NTLP on consecutive patients who present to participating health facilities during the 14-month study period (8 months of patient enrolment + 6 months of follow-up) and meet eligibility criteria. Implementation will be assessed using surveys and quantitative process metrics derived from routine clinical data and the 99DOTS server.

## Study population

*Eligibility Criteria*

1. *Site-level Inclusion Criteria*
   1. Diagnosed >10 PTB patients/month in 2017
   2. Not located within Kampala District
   3. Located within 225km of Kampala city
   4. PTB treatment success rate in 2016 of <80%
2. *Site-level Exclusion Criteria*
   1. Do not agree to participate in the study
3. *Patient-level Inclusion Criteria*
   1. Initiating Category I treatment for active pulmonary TB at a study facility during the study period
4. *Patient-level Exclusion Criteria*
   1. Diagnosed with extra-pulmonary TB
   2. Diagnosed with or referred for evaluation of drug-resistant TB
   3. Does not own or have access to a mobile phone with SMS capabilities

In addition, patients age <18 years, changed to Category II-IV treatment or transferred to another facility to complete treatment will be excluded from the primary analysis of all outcomes.

### ***Eligibility criteria for adoption, implementation, and costing studies (Aims 2 & 3)***

*Patient Interviews*: Interviews will be conducted with a convenience sample of patients eligible for the stepped-wedge randomized trial.

*Provider Interviews and Time-and-Motion Studies*: Providers at each study site who are (a) aged ≥18 years; (b) employed by the DTU; and (c) involved in the conduct or supervision of health facility work related to treatment and management of TB will be included.

*Settings and Locations*

Potential study sites were identified from a list of Uganda NTLP-affiliated TB treatment facilities. Study staff reviewed 2016 and 2017 treatment data reported to the Uganda NTLP to identify health facilities that meet eligibility criteria (based on numbers of patients tested and treated for TB), focusing on those within 225 km of Kampala for study feasibility purposes. We reviewed 2016 PTB treatment outcome and 2017 case finding data for all 1514 DTUs registered with NTLP. First-line anti-TB drugs are not available in the country other than through the NTLP (i.e., if diagnosed elsewhere, patients must be referred to a government or private facility registered with NTLP for TB treatment). Our review indicated that 79 of 1514 DTUs diagnosed and treated >=10 patients/month. Of these, 17 were in Kampala District, 31 were located greater >225km from Kampala City, and 8 had treatment success rate >80%. Thus, 23 DTUs met eligibility criteria and we selected 18 of these in consultation with NTLP. Study staff then obtained permission from the Uganda NTLP Director to visit health facilities to assess interest in study participation. During site visits, project staff met with the DHO or Hospital Director to inform him or her about the project. Key study procedures and expectations of participating sites were discussed using a standardized script. All 18 sites agreed to participate in the study.

## Interventions

*Intervention arm*: The 99DOTS-based strategy seeks to facilitate TB treatment completion by addressing barriers to traditional facility- or community-based treatment via DOT. Importantly, the 99DOTS-based strategy provides a higher level of treatment supervision than what occurs in routine care. As further described below, it includes the following core components:

| **Component** | | **Barrier addressed** |
| --- | --- | --- |
| 1 | Daily dosing confirmation via toll-free phone calls | Addresses high cost of clinic visits for patients, lack of real-time information for providers on patient adherence to medications. |
| 2 | Daily dosing reminders via automated SMS or pre-recorded phone calls | Addresses high cost of clinic visits for patients and assists with memory and planning processes known to be importance to adherence. |
| 3 | Weekly check-in via automated two-way SMS or interactive voice response phone calls | Addresses lack of social support and feeling of isolation during TB treatment; shown to be effective in other contexts at increasing connection with CHW and reducing social isolation. |
| 4 | Differential management protocol | Addresses limited time and resources among DTU staff and the need to focus on non-adherent patients. |

*Control arm*: TB treatment units in Uganda use a mix of facility- and community-based approaches to DOT.[^2^](#_ENREF_1) Most TB patients are asked to name a treatment supporter and are provided with a 2-week supply of medicines in the intensive phase (first two months) and a one-month supply of medicines in the continuation phase (second four months). Patients take their medicines at home (with or without observation by a treatment supporter) and are expected to return to clinic bi-monthly (intensive phase) or monthly (continuation phase) to check on side effects and obtain refills. At each refill visit, health center staff also assess adherence via patient self-report. Health center staff are supposed to call or visit patients who do not return for refills.

## Outcomes

*Definitions used to assess study outcomes*

- ***Number treated***: Number of eligible patients identified over a defined 8-month enrollment period through review of the NTLP Treatment register at each study site (includes patients treated for TB without undergoing any sputum testing). Patients started on treatment during the calendar month in which the 99DOTS-based strategy was introduced at each site will not be counted.
- ***Date treatment started***: Treatment start date recorded in the NTLP Treatment register and/or 99DOTS server. If discrepant, the NTLP Treatment register will be considered the primary source.
- ***Number enrolled on 99DOTS****:* Number of eligible patients entered into the 99DOTS registration system beginning on the 1^st^ day of the calendar month following the calendar month during which the 99DOTS-based strategy was introduced at each site through the end of the 8-month enrollment period.
- ***Date registered on 99DOTS***: Date of registration in 99DOTS as recorded in the 99DOTS server.
- ***Number treated successfully***: Number treated and with a treatment outcome of cured or completed entered into the Unit Treatment register, District Treatment Register, or Unit Treatment Register where patient was transferred.
- ***Number lost to follow***-***up***: Number treated and with a treatment outcome of lost to follow-up entered into the Unit Treatment register OR Unit Treatment Register where patient was transferred AND not recorded as having completed treatment or died in District TB Register.
- ***Number persisting on treatment***: Number treated and documented as having completed at least 60 doses of treatment without being lost to follow up. These 60 doses do not need to be consecutive. Data will be obtained from the treatment register. If a patient came to pick up their Month 3 continuation phase drugs, we will assume that they completed the first 60 doses.
- ***Number of scheduled doses confirmed by phone call***: Number of doses recorded by patient phone call to 99DOTS. This does not include manual doses programmed by a health care worker.
- ***Number of daily SMS sent by 99DOTS***: Number of daily medication reminders sent by 99DOTS, as recorded by the 99DOTS server. Each patient on the intervention should receive one SMS for each day of prescribed treatment.
- ***Number of daily SMS received on patient handset***: Number of daily medication reminder SMS successfully delivered, as recorded by 99DOTS server. This does not guarantee that the patient read the message, only that it was delivered.
- ***Number of weekly IVR calls to which patients send a response***: Number of weekly IVR calls to which patients respond with either 1 (feeling well) or 2 (not feeling well), recorded by the 99DOTS server.
- ***Number of weekly IVR calls received on patient handset***: Number of weekly IVR calls that are successfully picked up by the patient, as recorded by Africa’s Talking.

*Primary Outcome (Effectiveness)*

| **Outcome** | **Numerator** | **Denominator** |
| --- | --- | --- |
| Proportion treated successfully | Number treated successfully | Number treated |

*Secondary Outcomes (Effectiveness and Cost-Effectiveness)*

| **Outcome** | **Numerator** | **Denominator** |
| --- | --- | --- |
| Proportion with persistence | Number persisting on treatment | Number treated |
| Proportion lost to follow-up | Number lost to follow-up | Number treated |
| Incremental cost per patient treated successfully | Incremental cost | Incremental number of patients successfully treated |

### *Implementation Outcomes*

| **Implementation period only** | | |
| --- | --- | --- |
| **Outcome** | **Numerator** | **Denominator** |
| Proportion enrolled on 99DOTS | Number enrolled on 99DOTS | Number treated |
| Proportion of scheduled doses confirmed by phone call | Number of scheduled doses confirmed by phone call | Number enrolled on 99DOTS (or number called?) |
| Proportion of daily SMS sent by 99DOTS platform | Number of daily SMS sent by 99DOTS | Number of total days on treatment for all patients enrolled on 99DOTS |
| Proportion of daily SMS received on patient handset | Number of daily SMS received on patient handset | Number of daily SMS sent by 99DOTS |
| Proportion of weekly IVR calls sent by 99DOTS platform | Number of weekly IVR calls sent by 99DOTS | Number of total days on treatment for all patients enrolled on 99DOTS |
| Proportion of weekly IVR calls received on patient handset | Number of weekly IVR calls received on patient handset | Number of weekly IVR calls sent by 99DOTS |
| Proportion of weekly IVR calls to which patients send a response | Number of weekly IVR calls to which patients send a response | Number of weekly IVR calls received on patient handset |

## Sample Size

*Sample size*

Based on pre-randomization data collected from January to July 2017, we estimate 1890 patients will be enrolled across 18 study sites over the 8-month enrolment period.

*Justification*

The study aims to demonstrate the superiority of the 99DOTS-based strategy. The sample size calculation uses formulae appropriate for stepped-wedge trials.[^1^](#_ENREF_1) The primary outcome is the proportion of patients treated successfully. A type I error of 5% and power of at least 90% is assumed. Based on 2017 data, the harmonic mean number of patients initiating treatment for drug-susceptible PTB per month across project DTUs is 15. Thus, we anticipate approximately 1890 patients will initiate treatment over the 8-month enrollment period (945 in the pre- and 945 in the post-implementation phases across DTUs). We will have 90% power to demonstrate that our strategy increases the proportion of patients treated successfully by 10% or more and 80% power to demonstrate a 9% effect (assumptions: alpha=0.05; ICC = 0.001 calculated using 2017 NTLP data for the 18 DTUs; pre-implementation treatment success = 51% based on 2017 NTLP data for the 18 DTUs; calculations performed using *steppedwedge* command in Stata 14).

## Blinding

The trial will be open-label for participants and researchers, as blinding of the assigned intervention is not feasible given intervention implementation at the health facility level. Where possible, the investigators and study staff will be masked to ongoing aggregated data by study. The trial data analysis team will have access to the aggregate data.

# Randomization

Sequence generation for stepped wedge trials refers to the order with which clusters cross over from control to intervention.^1^ In our trial, eligible health facilities (N=18) will be randomly assigned to one of the six sequences using a simple, unrestricted two-stage process. This process will occur during a public ceremony for randomization held in Kampala, Uganda. Facility in-charges, district health officers, and representatives from NTLP will attend. The two stages of randomization will be as follows: *first*, health facilities will be assigned into clusters of equal size; and *second*, clusters will be randomly assigned into the sequence order in which they will switch to the intervention.

In the first stage, health facilities will be randomly assigned into six groups of three using a simple drawing. Each facility will have a printed name tag of equal size and color which will be placed into a non-see through bag and shuffled. 18 balls of the same size and color labelled A-F (x3) will be placed into a second non-see through bag, and also shuffled. A health facility name tag was randomly drawn from the first bag, and a representative from that facility will then draw a ball from the second bag, indicating which cluster group (A-F) they will be allocated to. This drawing will be performed 18 times, until both bags are empty and six groups of equal size (3 facilities each) are formed.

The second stage of randomization will generate the sequence in which group clusters A-F will switch from control to intervention. Six balls of the same size and color labelled 1-6 will be placed into a non-see through bag. Each ball corresponding to the month a group will switch to the intervention (1 = January, 2 = February, etc). A representative from each group A-F will random select a ball from the bag, and this will represent the group’s order within the sequence.

Clusters will be enrolled by local study staff, and eligible individual patients will be included in clusters through complete enumeration. District health officers provide administrative authority and consent to health facility participation prior to randomization. Consent is documented in a signed Memorandum of Understanding between the district and the National Tuberculosis and Leprosy Control Program (NTLP), endorsed by the Chief Administrative Officer, the District Health Officers, and the Program Manager. A copy of the agreement is provided to the facility In-change at each health facility.

## Statistical Methods

4.1.1 *General analyses principles*

In stepped-wedge trials (SWTs) clusters get switched from the control exposure to the intervention exposure according to a random sequence. Thus clusters spend varying time periods in the control condition before switching to intervention exposure. From the day a cluster is introduced to the intervention, a period of time elapses before effective exposure to the intervention can be plausibly and confidently assumed to have commenced. This transition interval is known as the buffer period. For analysis, the timing of the intervention exposure commences at the expiry of the buffer period. In this study, 3 clusters are trained and enroll their first patient under the intervention condition on different dates during a given calendar month (typically during the first, second and third week of the month). For purposes of standardization, the start of effective intervention exposure for all three clusters will be considered the first day of the next calendar month. This standardization will help fix the slices and improve statistical efficiency for the within-period analyses. We will conduct both within-period and between-period analyses. We will conduct multilevel random effects modelling to estimate the within-period and between-period intervention effects using a cluster-period mixed effects model.^3,4^ Analyses will be performed using Stata version 14/15. Given that all our outcomes (primary, secondary and implementation) are binary in nature, will use mixed effects (random intercept and random slope) logitmodels using *melogit* and *meqrlogit* (the new version of *xtmelogit*) commands.

Based on these general analysis principles, the specific analyses to be conducted are described below.

Step 1:  ***Explorative Data Analysis***

1. **Dataset assembly, cleaning and creation of new variables**

The TB treatment register data will be extracted from REDCap, transferred to Stata, and re-arranged into working analytic files. The data will be downloaded in wide format with one row per patient. Working analytic datasets will checked, each variable at a time for completeness, logical, range and consistency accuracy. Analysis variables will be generated following the definitions above for: period (intervention, control, buffer), month of treatment start, treatment success, lost to follow up, persistence, and conversion.

To determine which patients should be included in per protocol analyses, TB treatment register data will be merged with the list of patients enrolled on 99DOTS from 99dots.org using site, TB unit no. year, and TB unit no. A binary variable will indicate whether a patient should be included in per protocol analyses. Per protocol analyses will exclude 1) patients who started treatment prior to the intervention start at their site who were enrolled on 99DOTS and 2) patients who started treatment after the intervention start at their site who were not enrolled on 99DOTS, and 3) patients who started treatment after the intervention start at their site and were enrolled on 99DOTS >28 days after treatment initiation.

At this stage we will generate time-slice and exposure variables including the control-intervention exposure defining variable (var), the cluster-month *var*, cluster-exposure *var*, exposure-month *var* and the exposure-time-cluster var. Time variables will exclude the buffer period defined in section 4.1.1 above.

There are 18 TB designated treatment units (DTUs) that were assigned into six groups (3 DTUs per group) and one group at a time was switched from routine care to 99DOTS-based strategy in a randomly assigned order. Cluster level covariates (outcomes, predictors and confounders) will be aggregates of individual level of patients initiating TB treatment during the study enrollment period. For every cluster, each outcome (primary, secondary and implementation) occurring during the study period will be assigned to that cluster, the exposure condition (control or intervention) and time period in which the patient started treatment (study month). For each group of clusters, a summary measure of the outcome, *Y_ij_* (for *i* exposure and *j* period) will be calculated as a mean (SD) for continuous variables and log odds for binary variables. From the *Y_ij_*s, we will then calculate the cluster-period summaries Ʃ*Y_ij_* /n, where n=number of months in *i* exposure, and estimate unadjusted period-specific intervention effect (*θ* ) based on these cluster-period summaries.

1. **Baseline assessment and trend analysis**

Baseline measures of all outcomes stated in section 3.4 above will be calculated in study month 1 when all the clusters are in control exposure; estimates will be made for each cluster and group of clusters. The 99DOTS-based strategy will be introduced at the first three clusters in January 2019. Month 1 will therefore cover November and December 2018 since many study sites are closed during the second half of December for the holiday season. We will check for balance/imbalance in baseline covariates in the first month of the study commencement i.e., the first month of the control exposure period when all the clusters are in control. Comparisons will be done between clusters in each of the six groups (intra-group) and between groups of clusters (inter-group) using *t-test* and *ANOVA* for continuous variables and *Chi-square test, Fisher’s test* and *Proportion test* for categorical ones. Inter-group comparisons will be based on aggregated data from individual clusters of each group.

Within-period analysis comprise period-specific assessments where aggregated outcomes of clusters in the control exposure are compared with those of clusters in intervention exposure during the same time period. While in between-period analyses, outcomes for each cluster or group of clusters are compared for when the same cluster(s) was/were in intervention versus control exposures i.e., cluster-specific control-intervention comparisons across different time periods.

Post-baseline measures will include control exposure and intervention exposure since groups of clusters will enter intervention exposure at varying times. For clusters still in the control exposure, each subsequent calendar month after month 1 before commencement of the buffer period will be analysed as post-baseline control period. As described above in section 4.1.1, the start of effective intervention exposure (*i.e.,* post buffer period) for all three clusters will be the first day of the calendar month following the month in which training took place.

Trend analyses based on monthly summary measures of the primary outcome will be conducted for each group of clusters for both control and intervention exposure periods. We will assess both change in level of the outcome at the time of switching (immediate effect) and change in slope (sustained effect). This check will identify pre-intervention levels (average) and slopes (rate of change with time) that could be extending to the intervention phase unchanged or their changes (magnitude and statistical significance). This is illustrated in the graphs below.

Figure 1: Change in average level of outcome Figure 2: Change in level with same slope of outcome

Figure 3: Change and no change in slopes of outcome Figure 4: Change in both level and slopes of outcome

As illustrated in the above four figures, a simple pre-and post-intervention comparison in the average level of the outcome may indicate a statistically significant difference when in actuality there is not difference in the slope between the two periods (figure 2) and such a simple comparison may lead to erroneous conclusions about the effect of the intervention. Pre-and post-intervention differences can occur in both the level and slope of outcome (figures 3 and 4). Since the study design is a one-way cross over that did not involve randomization of clusters to receive the intervention or remain in control at the stage of interruption i.e. all clusters switched from control to intervention as opposed to some remaining in control at the time of exposure interruption, we will fit single group series models to assess the population average level and slope changes. For these analysis, we will fit models with outcomes of cluster-period summaries calculated as indicated in paragraph 2 of step 2 above, using cluster-time and time-exposure interaction terms. In the models (*melogit and meqrlogit since the outcomes are all binary and estimates will be on log odds scale*), a given outcome denoted *Y_t_* represents the aggregated outcome within each time slice t, calculated from *Y_ijk_* (for *i* exposure, *j* period and cluster *k*). If there is not a sustained change in slope over the entire period, we will employ linear spline modelling (with inflection points at various months in the intervention exposure period to determine how far the effect increases before it tapers off or if the increase in the intervention effect is different within different periods. There four possible ways in which the analysis results may turn out namely: 1) No change in level, no change in slope; 2) No Change in level, change in slope; 3) Change in level, no change in slope; and 4) change in both level and slope.

Logit(Y_t_) = β_0_ + β_1_T_t_ + β_2_X_t_ + β_3_X_t_T_t_ + β_4_C_k_X_t_T_t_ + u_i_  …………………………………………………. Equation 1

Where,

*Y_t_* = aggregated outcome within each time slice t, calculated from *Y_ijk_* (for *i* exposure, *j* period and cluster *k*)

*T_t_* = the time since the start of the study,

*X_t_* = indicator variable representing the exposure conditions with control coded 0 and intervention coded 1.

*X_t_T_t_* is an interaction term.

β_4_C_k_X_t_T_t_ is the cluster-exposure-time interaction term

*β_0_* represents the intercept or starting level of the outcome variable.

*β_1_* is the slope of the outcome in the pre-intervention (control) period.

*β_2_* represents the difference in the level of the outcome between pre-and post-intervention periods.

*β_3_* represents the diﬀerence between pre-intervention and post-intervention slopes of the outcome.

*Β_4_* represents the group of cluster diﬀerence between pre-intervention and post-intervention slopes of the outcome

***Intervention Effect Analysis***

The primary outcome is the proportion of TB patients treated successfully measured as number treated successfully divided by the number treated. Successful treatment is taken as a treatment outcome of cured or completed as stated in the Unit Treatment register. This proportion will be calculated for each cluster and group of clusters for each study time-exposure slice. The effect of the intervention will be evaluated by comparing adjusted slope differences in the primary outcomes between the intervention period and the control period, using random intercept and random slope (mixed) logit models. Analysis will be done in Stata using *melogit* and *meqrlogit* commands.

The effect of the intervention will be evaluated by comparing adjusted slope differences in the primary outcomes between the intervention period and the control period, using random intercept and random slope (mixed) logit models Analysis will be done in Stata using *melogit* and *meqrlogit* commands. The random intercept, and random slope models take the general form of:

Y_ijk_ = μ + β_j_ + θX_ij_ + u_i_ + e_ijk_, …………………………………………………………………. Equation 2

where y_ijk_ is the outcome of individual *k* in period *j* from cluster *i*, μ is the mean outcome in the first period, β_j_ is the difference between period j and the first period with β_1_=0, θ is the intervention effect, X_ij_ is 1 if cluster i received the intervention in period j and 0 otherwise, u_i_ is a random effect for cluster, and e_ijk_ is the within-cluster variability.

In SWT, the outcomes to be compared/assessed are at cluster level hence the general model form will be:

*Y_t_* = μ + β_j_ + θX_ij_ + u_i_ + e_ijk_, …………………………………………………………………... Equation 3

where *Y_t_* = aggregated outcome within each time slice t, calculated from *Y_ijk_* (for *i* exposure, *j* period and cluster *k*). We will adjust for unbalanced time independent variables at baseline and confounders. The proportion of the outcome variable will be calculated for each cluster and group of clusters for each study time-exposure slice. We will assess both change in level of the outcome at the time of switching (immediate effect) and change in slope (sustained effect) by fitting models with following interactions terms; cluster-month, cluster-exposure, exposure-month, and the exposure-time-cluster. These cross-product terms will enable us to assess within-period differences between clusters in control and intervention exposures, between-period differences in the same cluster or group of clusters acting as own controls, secular trends and the differences in level and slopes between the control and intervention exposures respectively. Models will be fit progressively from unadjusted to adjusted ones assessing; i) time effect only; ii) treatment effect only; iii) cluster effect only; iii) time-cluster interaction; vi) time-treatment interaction; and time-cluster-treatment interaction.

For between period (horizontal) comparisons, comparative analysis is done at cluster level hence the general model form is:

logit(*Y_jt_)* = α + βx*_jt_* + Ʃ*ץ_t_*z*_t_*+ u*_j_* ……………………………………………………………….... Equation 4

where *j* indexes the clusters, *t* indexes the steps, x*_jt_* indicates a cluster and exposure; is coded 0 when the *j*th cluster is in control exposure and 1 when it is in the intervention exposure, *β* represent the intervention effect, *ץ_t_* represents the time-specific effects, z*_t_* the steps and u*_j_* represents the cluster level random effects (within-cluster correlation).

Within-period (verical) comparisons are done by fitting a model as in equation 4 but with covariates of month, exposure, cluster-month, cluster-exposure, and the interaction term of exposure-time-cluster. As already indicated, we will use Stata *melogit and meqrlogit* since the outcomes are all binary and estimates will be on log odds scale; *meqrlogit* provides for more model convergence than *melogit*.

***Populations and subgroups to be analysed***

*Populations*

Intention-to-treat (ITT): The ITT population will include all health centers and all eligibile patients who were enrolled in TB treatment at these health centers. Patients will be included in the analysis in the intervention/control assignment of their health center at the time of treatment initiation.

Per-Protocol (PP): The PP population will include all health centers; however it will only include the eligible patients who received the appropriate intervention.The analysis will include eligible patients who received the standard of care during control periods at health centers assigned to control, and elibile patients who received the intervention during intervention periods at health centers assigned to the intervention. Patients who should have received the intervention by randomization but instead received the standard of care and patients who started treatment during the control period but received the intervention will be dropped.

*Subgroup analyses*

Three types of subgroups will be analysed using both ITT and PP populations.

1. Gender: All eligible patients at randomized health centers will be divided into two groups by gender and analyzed separately.

2. HIV Status: All eligible patients at randomized health centers will be divided into two groups by HIV status (HIV-positive and HIV-negative/unknown) and analysed separately.

3. Site: Eligible patients at each of the 18 health centers will be analysed separately.

***Sensitivity analyses of primary outcomes***

Sensitivity analyses will be performed to assess the robustness of our findings with respect to the following factors:

1. Treatment outcomes for patients lost to follow up

2. The staged intervention roll out

3. Intervention timing and buffer period

4. Analysis method^5^

***Analysis of secondary outcomes***

Secondary outcomes will be analysed in the same manner as the primary outcome.

**Table 1. 99DOTS Randomization and Enrolment Schedule**

|  | **Month** | | | | | | | |
| --- | --- | --- | --- | --- | --- | --- | --- | --- |
|  | 1 | 2 | 3 | 4 | 5 | 6 | 7 | 8 |
| Group 6 |  |  |  |  |  |  |  |  |
| Group 5 |  |  |  |  |  |  |  |  |
| Group 4 |  |  |  |  |  |  |  |  |
| Group 3 |  |  |  |  |  |  |  |  |
| Group 2 |  |  |  |  |  |  |  |  |
| Group 1 |  |  |  |  |  |  |  |  |

Routine care

Switch to 99DOTS

99DOTS implementation

**References**

- 1. Hemming Karla, Taljaard Monica, McKenzie Joanne E, Hooper Richard, Copas Andrew, Thompson Jennifer A, et al. Reporting of stepped wedge cluster randomised trials: extension of the CONSORT 2010 statement with explanation and elaboration. *BMJ* 2018; 363 :k1614
  2. Ministry of Health. Manual for Management and Control of TB and Leprosy 2017. Republic of Uganda. <http://library.health.go.ug/publications/service-delivery-diseases-control-prevention-communicable-diseases/tuberculosis/manual>. Published 2017.
  3. Thompson J.A, Davey C, Fieiding K, Hargreaves J.R, Hayes R.J. Robust analysis off stepped wedge trials using cluster-level summaries periods. Statistics in Medicine 2018;37:2487-2500.
  4. Hemming K, Taljaard M, Forbes A. Analysis of cluster randomized stepped wedge trials with repeated cross-sectional samples. Trials (2017) 18:101 DOI 10.1186/s13063-017-1833-7.
  5. Thompson, JA (2018) Improving the Design and Analysis of Stepped-Wedge Trials. PhD thesis, London School of Hygiene & Tropical Medicine. DOI: https://doi.org/10.17037/PUBS.04647855
