# Supplementary material for: Digital adherence technology for tuberculosis treatment supervision: A stepped-wedge cluster-randomized trial in Uganda
Source: PLoS Med. 2021 May 6;18(5):e1003628. doi: 10.1371/journal.pmed.1003628 (PMC8136841; doi:10.1371/journal.pmed.1003628)
Supplement: S1 Table — ITT, intention-to-treat; PP, per protocol. *Primary outcome. **Secondary outcome. †Adjusted for time (trial month, discrete variable), sex, HIV status, disease class (bacteriologically confirmed versus clinically diagnosed), and TB type (new versus retreatment) as fixed effects and site as a random effect. ^Proportion difference calculated as proportion in intervention period minus that in the control period. ¶p-Value for adjusted intervention effect. (DOCX) [file pmed.1003628.s007.docx]

|  | **N** | **Unadjusted Proportion**  **n/N (%)** | | **Effect Estimate** | | |
| --- | --- | --- | --- | --- | --- | --- |
|  |  | **Control  Period** | **Intervention  Period** | **Adjusted Proportion Difference^†^**^^^ **(95% CI)** | **Adjusted Odds**  **Ratio^†^ (95% CI)** | **P value**^¶^ |
| **Treated successfully*** | | | | | | |
| ITT | 2,224 | 814/1140 (71.4%) | 799/1084 (73.7%) | 2.68 (-3.43, 8.78) | 1.16 (0.83, 1.61) | 0.39 |
| PP | 1,667 | 770/1088 (70.8%) | 502/579 (86.7%) | 14.95 (8.18, 21.72) | 2.62 (1.66, 4.12) | <0.001 |
| **Completed intensive phase**** | | | | | | |
| ITT | 2,224 | 899/1140 (78.9%) | 884/1084 (81.6%) | 3.69 (-1.61, 9.00) | 1.28 (0.90, 1.84) | 0.17 |
| PP | 1,667 | 851/1088 (78.2%) | 530/597 (91.5%) | 14.01 (9.16, 20.67) | 3.61 (2.13, 6.12) | <0.001 |
| **Not lost to follow-up**** | | | | | | |
| ITT | 2,224 | 993/1140 (87.1%) | 955/1084 (88.1%) | 1.09 (-3.45, 5.63) | 1.11 (0.72, 1.71) | 0.64 |
| PP | 1,667 | 942/1088 (86.6%) | 562/579 (97.1%) | 11.58 (6.30, 16.87) | 5.77 (2.65, 12.56) | <0.001 |
