# Supplementary material for: Digital adherence technology for tuberculosis treatment supervision: A stepped-wedge cluster-randomized trial in Uganda
Source: PLoS Med. 2021 May 6;18(5):e1003628. doi: 10.1371/journal.pmed.1003628 (PMC8136841; doi:10.1371/journal.pmed.1003628)
Supplement: S1 Trial Protocol — (DOC) [file pmed.1003628.s009.doc]

**From Directly-Observed Therapy (DOT) to Digital Adherence Technology (DAT) for TB Treatment – A Pragmatic, Implementation Evaluation**

**Sponsored by:**

TB REACH

**Unique Protocol ID:**

1180

**Principal Investigator:**

Adithya Cattamanchi MD, MAS

**Draft or Version Number:**

*Version 2.0*

**Day Month Year**

November 16, 2018

**Statement of Compliance**

The study will be carried out in accordance with Good Clinical Practice (GCP) as required by the following:

- *U.S. Code of Federal Regulations applicable to clinical studies (45 CFR 46)*
- *ICH GCP E6*
- *Completion of Human Subjects Protection Training*
- *NIH Clinical Terms of Award*

*Refer to: [http://www.hhs.gov/ohrp/humansubjects/guidance/45cfr46.htm#46](http://www.hhs.gov/ohrp/humansubjects/guidance/45cfr46.htm#46)*

[*http://www.fda.gov/cder/guidance/959fnl.pdf*](http://www.fda.gov/cder/guidance/959fnl.pdf)

[*http://grants.nih.gov/grants/guide/notice-files/NOT-OD-01-061.html*](http://grants.nih.gov/grants/guide/notice-files/NOT-OD-01-061.html)

[*http://cme.cancer.gov/c01/*](http://cme.cancer.gov/c01/)

**Signature Page**

The signature below constitutes the approval of this protocol and the attachments, and provides the necessary assurances that this study will be conducted according to all stipulations of the protocol, including all statements regarding confidentiality, and according to local legal and regulatory requirements and applicable US federal regulations and ICH guidelines.

Uganda Study Site Principal Investigator:*

Signed:________________________________ Date:_____________________

Achilles Katamba, MBChB, PhD

Overall Study Principal Investigator:*

Signed:________________________________ Date:_____________________

Adithya Cattamanchi, MD, MAS

Table of Contents

[Overview 5](#__RefHeading___Toc530141227)

[Key Roles 6](#__RefHeading___Toc530141228)

[Table 1. Timeline of Activities 7](#__RefHeading___Toc530141229)

[Background 8](#__RefHeading___Toc530141230)

[Background information 8](#__RefHeading___Toc530141231)

[Theoretical basis for strategy 9](#__RefHeading___Toc530141232)

[Objectives 10](#__RefHeading___Toc530141233)

[Methods/Design 11](#__RefHeading___Toc530141234)

[Overview 11](#__RefHeading___Toc530141235)

[Target Setting and Study Population 11](#__RefHeading___Toc530141236)

[Eligibility criteria for the stepped-wedge implementation study (Aim 1) 11](#__RefHeading___Toc530141237)

[Eligibility criteria for adoption, implementation, and costing studies (Aims 2 & 3) 11](#__RefHeading___Toc530141238)

[Recruitment 12](#__RefHeading___Toc530141239)

[Health centers 12](#__RefHeading___Toc530141240)

[Participants for stepped-wedge implementation study (Aim 1) 12](#__RefHeading___Toc530141241)

[Participants for 99DOTS design interviews (Aim 2) 12](#__RefHeading___Toc530141242)

[Participants for adoption, implementation, and costing studies (Aims 2 & 3) 12](#__RefHeading___Toc530141243)

[Routine care and Intervention strategy 13](#__RefHeading___Toc530141244)

[Routine care 13](#__RefHeading___Toc530141245)

[Intervention strategy 13](#__RefHeading___Toc530141246)

[Randomization 14](#__RefHeading___Toc530141247)

[Procedures 14](#__RefHeading___Toc530141248)

[Pre-implementation site visit 14](#__RefHeading___Toc530141249)

[Health center intervention strategy training 15](#__RefHeading___Toc530141250)

[Patient-level data collection 15](#__RefHeading___Toc530141251)

[Process metric data collection 15](#__RefHeading___Toc530141252)

[Patient and provider interviews to refine design of 99DOTS 15](#__RefHeading___Toc530141253)

[Patient and provider interviews to identify factors influencing adoption and implementation of the intervention strategy 16](#__RefHeading___Toc530141254)

[Study Instruments 16](#__RefHeading___Toc530141255)

[Outcomes 17](#__RefHeading___Toc530141256)

[Definitions 17](#__RefHeading___Toc530141257)

[I. Primary Outcome (Effectiveness) 17](#__RefHeading___Toc530141258)

[II. Secondary Outcomes (Effectiveness and Cost-Effectiveness) 17](#__RefHeading___Toc530141259)

[III. Implementation Outcomes 18](#__RefHeading___Toc530141260)

[Data Management 18](#__RefHeading___Toc530141261)

[Statistical Analysis 19](#__RefHeading___Toc530141262)

[Aim 1 19](#__RefHeading___Toc530141263)

[Aim 2 19](#__RefHeading___Toc530141264)

[Aim 3 19](#__RefHeading___Toc530141265)

[Sample Size Considerations 20](#__RefHeading___Toc530141266)

[Aim 1 20](#__RefHeading___Toc530141267)

[Aim 2 20](#__RefHeading___Toc530141268)

[Aim 3 20](#__RefHeading___Toc530141269)

[Ethical Considerations 21](#__RefHeading___Toc530141270)

[Potential risks to participants 21](#__RefHeading___Toc530141271)

[Protection against risk 21](#__RefHeading___Toc530141272)

[Data protection 22](#__RefHeading___Toc530141273)

[Potential benefits of the study to participants and society 22](#__RefHeading___Toc530141274)

[Dissemination 22](#__RefHeading___Toc530141275)

[Study governance 22](#__RefHeading___Toc530141276)

[Abbreviations 23](#__RefHeading___Toc530141277)

[Competing interests 24](#__RefHeading___Toc530141278)

[References 25](#__RefHeading___Toc530141279)

# Overview

| **Title** | From Directly-Observed Therapy (DOT) to Digital Adherence Technology (DAT) for TB Treatment – A Pragmatic Implementation Evaluation |
| --- | --- |
| **Target Population** | Adults undergoing evaluation for pulmonary TB |
| **Sites** | 18 TB treatment units in Uganda (3 health centers, 15 hospitals) |
| **Study Design** | Pragmatic, stepped-wedge implementation study |
| **Study Duration** | 2 years |
| **Primary Objective** | **To determine whether a 99DOTS-based strategy improves TB treatment outcomes compared to routine care**   - *Strategy:*  1. Confirmation of dosing via toll-free phone calls using 99DOTS 2. SMS dosing reminders to patients delivered via 99DOTS 3. Weekly two-way check-in via SMS or interactive voice response using 99DOTS 4. Differential management based on dosing history and response to weekly check-in.  - *Standard-of-care*: routine community- or facility-based DOTS treatment |

# Key Roles

**Principal Investigator**

Adithya Cattamanchi, MD, MAS

Associate Professor of Medicine, University of California San Francisco

San Francisco General Hospital

1001 Potrero Ave, Room 5K1

San Francisco, CA 94110

Phone: +1-415-206-5489

Fax: +1-415-695-1551

adithya.cattamanchi@ucsf.edu

**Uganda Principal Investigator**

Achilles Katamba, MBChB, MSc, PhD

Senior Lecturer, Makerere University College of Health Sciences

Upper Mulago Hill Road

Kampala, Uganda

Phone: +256-(0)414-530-692

Fax: +256-(0)414-540-524

axk95@case.edu

**TB REACH Technical Officer**

Amera Khan, MPH

Technical Officer, Stop TB Partnership

Phone: +41 79 521 78 20

Stop TB Partnership Secretariat

Chemin du Pommier 40

1218 Le Grand-Saconnex

Geneva, Switzerland

amerak@stoptb.org

# Table 1. Timeline of Activities

|  | **YEAR 1** | | | | **YEAR 2** | | | |
| --- | --- | --- | --- | --- | --- | --- | --- | --- |
|  | **2018** | | **2019** | | | | **2020** | |
|  | **Q3** | **Q4** | **Q1** | **Q2** | **Q3** | **Q4** | **Q1** | **Q2** |
| **Enroll study sites and conduct TB guidelines training** |  |  |  |  |  |  |  |  |
| **Collect baseline (pre-implementation) data** |  |  |  |  |  |  |  |  |
| TB focal person survey |  |  |  |  |  |  |  |  |
| Patient-level outcomes from TB registers (2017) |  |  |  |  |  |  |  |  |
| Human-centered design, Pre-implementation assessment |  |  |  |  |  |  |  |  |
| **Assign sites to strategy and routine care arms** |  |  |  |  |  |  |  |  |
| Stepped-wedge randomization |  |  |  |  |  |  |  |  |
| Randomization unveiling meeting |  |  |  |  |  |  |  |  |
| **Prepare for launch of 99DOTS** |  |  |  |  |  |  |  |  |
| Design, print, and ship initial IP and CP 99DOTS envelopes |  |  |  |  |  |  |  |  |
| Finalize 99DOTS logistics & technical components |  |  |  |  |  |  |  |  |
| Conduct training of trainers on 99DOTS |  |  |  |  |  |  |  |  |
| Adapt and finalize patient & provider training materials |  |  |  |  |  |  |  |  |
| **Implement the strategy** |  |  |  |  |  |  |  |  |
| Switch health centers to 99DOTS (3 DTUs/month) |  |  |  |  |  |  |  |  |
| Collect patient data from treatment registers & cards |  |  |  |  |  |  |  |  |
| **Adapt Human Centered Design 99DOTS components** |  |  |  |  |  |  |  |  |
| HCD post-implementation interviews of patients enrolled in month 1 |  |  |  |  |  |  |  |  |
| Finalize IP design 2 +/- CP design 2 |  |  |  |  |  |  |  |  |
| Obtain feedback from CP patients enrolled in month 1 & 2 |  |  |  |  |  |  |  |  |
| Finalize CP design 2 or 3 |  |  |  |  |  |  |  |  |
| **Assess strategy adoption and implementation (strategy sites)** |  |  |  |  |  |  |  |  |
| Collect process metric data |  |  |  |  |  |  |  |  |
| Conduct CFIR post-implementation patient and provider interviews |  |  |  |  |  |  |  |  |
| **Assess cost-effectiveness and impact** |  |  |  |  |  |  |  |  |
| Interview patients & providers on costs |  |  |  |  |  |  |  |  |
| Conduct time-and-motion & budgetary analyses at health centers |  |  |  |  |  |  |  |  |

# Background

## Background information

Tuberculosis (TB) is now the leading infectious cause of death worldwide and incomplete patient treatment continues to be an obstacle to the elimination of this disease. Worldwide treatment success rates for drug-susceptible TB are below the 90% target for the year 2025 published in the World Health Organization’s (WHO) 2017 global tuberculosis report.^1^ Promoting treatment adherence will be critical to improve treatment success, slow multidrug resistance, and progress towards TB eradication. Since 1993, DOTS has been the WHO-recommended strategy for TB treatment. A central aspect of DOTS has been a health worker observing the patient when he or she swallows each dose of anti-TB medication (directly observed therapy, DOT). However, major obstacles to implementation success of DOT include: 1) high costs faced by patients to attend clinic visits; 2) lack of adequate social support and isolation experienced by patients; 3) lack of real-time information on medication adherence to guide clinical care; and 4) inability of providers to focus attention on patients most in need of additional treatment support. Novel, patient-centered approaches are needed to improve treatment adherence and facilitate treatment supervision.

Recently, there has been increasing interest in digital technologies for TB treatment, especially though the use of mobile phones and short messaging service (SMS). Such technologies were mentioned for the first time in the WHO 2017 treatment guidelines for drug-susceptible TB^3^, but received only a conditional recommendation due to very low quality of evidence. In the first randomized trial of digital adherence technologies, Liu et al found that reminders from medication monitors but not SMS improved medication adherence, but SMS reminders did not. However, there were no significant differences in poor treatment outcomes (treatment failure, death or loss to follow-up).^4^ Similarly, in a randomized trial in Cameroon, Bediang et al found that SMS reminders did not increase TB treatment success and cure rates, and that loss to follow-up rates were high at around 32-34% in both the intervention and control groups.^5^ Finally, Mohammed et al also found that daily SMS reminders resulted in no significant differences in treatment outcomes in a randomized trial in Pakistan, hypothesizing that enhanced usage of SMS for other types of reminders and greater off-site support could improve uptake.^6^ Additional research is therefore needed to determine if augmented approaches to digital adherence monitoring can further improve TB treatment outcomes.

99DOTS (Everwell Health Solutions, India) is a simple and low-cost ($4-6/patient at scale) digital adherence technology that involves patients calling toll-free phone numbers – ordered in a pattern that is not predictable by patients – underneath pills in blister packs. When patients remove scheduled medication doses from the blister pack, the phone numbers are revealed, enabling patients to make toll-free calls to self-report medication dosing. Clinic staff can access adherence data for individual patients in real-time through a web dashboard and mobile phone application. SMS reminders (to patients for dosing and to providers to follow-up on patients who have missed doses) and interactive two-way messaging are also core features of the platform. 99DOTS thus enables real-time identification of patients who miss doses for further follow-up (e.g., targeted adherence counseling, home visits, etc.) and the use of supportive messaging other than reminders through an open-source platform.

The overall objective of the DOT to DAT implementation study is to evaluate the effectiveness, implementation and costs of a 99DOTS-based strategy to improving TB treatment adherence. Our hypotheses are that the 99DOTS-based strategy will lead to higher treatment completion, have high uptake among patients and providers, and be cost-effective. To test our hypotheses, we will conduct a pragmatic, stepped-wedge evaluation at 18 TB designated treatment units (DTUs) in Uganda in which the units will be assigned to switch from routine care to the 99DOTS-based strategy in a random order.

## Theoretical basis for strategy

Documented bottlenecks to TB treatment adherence categorized in accordance with the PRECEDE model for design of multi-faceted health promotion interventions include:

***Predisposing Factors:***

- High costs (direct + indirect) of clinic visits, limiting patients’ ability and motivation to complete treatment
- Lack of social support and feeling of isolation during TB treatment

***Enabling Factors:***

- Inability of DTU staff to focus limited time and resources on non-adherent patients

***Reinforcing factors:***

- Lack of real-time information on patient adherence to medications

The 99DOTS-based strategy was designed in consultation with the Uganda NTP to target these bottlenecks. In addition to its basic features (dosing verification through toll-free phone calls to hidden numbers for each dose), we propose using the 99DOTS platform to 1) provide external cues and facilitate memory and planning through daily SMS reminders to patients; 2) address support barriers, enhance self-efficacy, and reduce concerns about consequences (i.e., side effects) through weekly check-in via two-way SMS or interactive voice response (weekly automated call to patients asking to press 1 if they feel fine, 2 otherwise) modeled after the WelTel program to facilitate ART adherence in Kenya^7^; and 3) facilitate differential management based on dosing history and response to weekly check-in. 99DOTS will provide a greater level of treatment supervision relative to routine care by enabling real-time monitoring of patient dosing history (as logged by toll-free phone calls made by patients to report they have ingested their daily medication dose). Per current practice, patients will be asked to return to clinic every 2 weeks for refills during the first 8 weeks of treatment. Subsequently, patients deemed to have taken >90% of doses will be asked at the 4- and 8-week visits if they prefer to return to clinic monthly or every 2 months, respectively. In contrast, patients who miss >2 consecutive doses, take <90% of doses overall, or do not respond to the weekly check-in will have enhanced follow-up (via a standardized protocol) through phone calls and, if no response, a home visit from a CHW. DTU staff will identify patients who require enhanced follow-up, as well as those eligible for more a more liberalized refill schedule, through the 99DOTS mobile phone app (and document whether follow-up was completed via the app). Thus, 99DOTS may enable health center staff to focus their time and resources on patients most in need of additional support.

# Objectives

**Aim 1: Determine whether a 99DOTS-based strategy improves TB treatment outcomes.**

Eighteen TB treatment units will be included in a pragmatic, stepped-wedge evaluation of the 99DOTS-based strategy. The primary outcome will be the proportion of patients completing treatment successfully. Key secondary outcomes will include persistence (proportion of patients remaining on treatment at 2 months), conversion (proportion of bacteriologically-positive patients who are smear-negative at 2 months) and proportion lost to follow-up.

**Aim 2: Evaluate reach, adoption and implementation of the 99DOTS-based strategy.**

During the post-implementation period at each site, we will assess the proportion of eligible patients registered on 99DOTS. We will use quantitative process metrics to assess adoption and implementation of each component of the 99DOTS-based strategy. We will also collect quantitative and qualitative data to identify key differences between high and low adoption of 99DOTS at both the health facility and patient levels.

**Aim 3: Evaluate the incremental costs, outcomes, and cost-effectiveness associated with 99DOTS as compared to the standard of care from the health system and patient perspectives.**

We will collect cost and operational data using time-and-motion (TAM) studies, staff and patient interviews, and analysis of budgetary and administrative records to assess whether 99DOTS is cost-effective and improves social and economic outcomes for patients being treated for TB.

# Methods/Design

## Overview

The study proposes to conduct a pragmatic, stepped-wedge implementation study to evaluate the effectiveness and implementation of the strategy relative to standard supervision of TB treatment. The effectiveness of the strategy will be assessed using routine data collected as part of mandatory reporting to the Uganda NTLP on consecutive patients who present to participating health centers during the 14-month study period (8 months of patient enrollment + 6 months of follow-up) and meet eligibility criteria. Implementation will be assessed using quantitative process metrics derived from routine clinical data and the 99DOTS server.

## Target Setting and Study Population

The target setting is TB treatment units in Uganda. The unit of randomization will be the TB treatment unit (N=18). The unit of analysis will be patients initiating TB treatment during the study enrollment period (estimated total 1890 patients over 8-months).

### ***Eligibility criteria for the stepped-wedge implementation study (Aim 1)***

1. *Site-level Inclusion Criteria*
   1. Diagnosed >10 PTB patients/month in 2017
   2. Not located within Kampala District
   3. Located within 225km of Kampala city
   4. PTB treatment success rate in 2016 of <80%
2. *Site-level Exclusion Criteria*
   1. Do not agree to participate in the study
3. *Patient-level Inclusion Criteria*
   1. Initiating Category I treatment for active pulmonary TB at a study facility during the study period
4. *Patient-level Exclusion Criteria*
   1. Diagnosed with extra-pulmonary TB
   2. Diagnosed with or referred for evaluation of drug-resistant TB
   3. Does not own or have access to a mobile phone with SMS capabilities

In addition, patients age <18 years, changed to Category II-IV treatment or transferred to another facility to complete treatment will be excluded from the primary analysis of all outcomes.

### ***Eligibility criteria for adoption, implementation, and costing studies (Aims 2 & 3)***

*Patient Interviews*: Interviews will be conducted with a convenience sample of patients eligible for the stepped-wedge implementation study.

*Provider Interviews and Time-and-Motion Studies*: Providers at each study site who are (a) aged ≥18 years; (b) employed by the DTU; and (c) involved in the conduct or supervision of health center work related to treatment and management of TB will be included.

## Recruitment

### ***Health centers***

Potential study sites were identified from a list of Uganda NTLP-affiliated TB treatment centers. Study staff reviewed 2016 and 2017 treatment data reported to the Uganda NTLP to identify health centers that meet eligibility criteria (based on numbers of patients tested and treated for TB), focusing on those within 225 km of Kampala for study feasibility purposes. We reviewed 2016 PTB treatment outcome and 2017 case finding data for all 1514 DTUs registered with NTLP. First-line anti-TB drugs are not available in the country other than through the NTLP (i.e., if diagnosed elsewhere, patients must be referred to a government or private facility registered with NTLP for TB treatment). Our review indicated that 79 of 1514 DTUs diagnosed and treated >=10 patients/month. Of these, 17 were in Kampala District, 31 were located greater >225km from Kampala City, and 8 had treatment success rate >80%. Thus, 23 DTUs met eligibility criteria and we selected 18 of these in consultation with NTLP. Study staff then obtained permission from the Uganda NTLP Director to visit health facilities to assess interest in study participation. During site visits, project staff met with the DHO or Hospital Director to inform him or her about the project. Key study procedures and expectations of participating sites were discussed using a standardized script. All 18 sites agreed to participate in the study.

### ***Participants for stepped-wedge implementation study (Aim 1)***

This is a pragmatic study that will evaluate outcomes of TB treatment. Thus, rather than recruiting participants, patient and implementation outcomes will be assessed during the pre- and post-implementation periods at each site using data available in routine TB Treatment registers and TB Treatment cards for consecutive patients who initiate TB treatment at participating health centers during the 8-month patient enrollment period and meet eligibility criteria (estimated N=1890 patients). Process outcomes will also be assessed using data related to patient phone calls/SMS messages and provider response to phone calls/SMS messages available on the 99DOTS server.

### ***Participants for 99DOTS design interviews (Aim 2)***

Interviews will be conducted to gain key insights into the user experience with 99DOTS to help further refine its design. Patient and provider interviews will be conducted at Month 3 and 5 of the study period. In Month 3, study staff will visit the first 3 sites that have implemented the 99DOTS-based intervention strategy. At each site, study staff will interview 5-10 patients (+/- their treatment supporter) and 2-3 key providers involved in delivering TB treatment services. Research staff will also aim to conduct at least one home visit with the permission of the patient after their interview. In Month 5, the team will visit 6 sites that have implemented the 99DOTS-based intervention strategy (all distinct from those visited in Month 3), and conduct patient and provider interviews as described for Month 3.

### ***Participants for adoption, implementation, and costing studies (Aims 2 & 3)***

*Patient Interviews* will be conducted at all sites to assess factors influencing adoption and implementation of the intervention strategy (N=5-10/site; 90-180 total) and at selected sites to assess patient costs associated with 99DOTS relative to standard of care (N=5-10/site at 6-8 sites; 30-80 total). DTU staff will assist with identification and recruitment of patients who are interested in participating in the interviews (from among eligible patients receiving treatment). Participation will be voluntary. Research staff will then contact interested patients in-person or by phone to review the verbal consent script, answer questions the patient may have, and administer the interview to consenting patients.

*Provider Interviews* will be conducted at all sites to assess factors influencing intervention adoption and implementation of the intervention strategy *(*N=5-10/site; 90-180 total). All eligible providers at each study site will be invited to participate. Participation will be voluntary and verbal consent will be obtained.

*Provider Time-and-Motion Studies* will be conducted at six sites (one per intervention wave) to assess time associated with activities related to TB treatment during both the control and intervention periods *(*N=3-5/site; 18-30 total). All eligible providers at each study site will be invited to participate. Participation will be voluntary and verbal consent will be obtained.

## Routine care and Intervention strategy

### ***Routine care***

TB treatment units in Uganda use a mix of facility- and community-based approaches to DOT.^2^ Most TB patients are asked to name a treatment supporter and are provided with a 2-week supply of medicines in the intensive phase (first two months) and a one-month supply of medicines in the continuation phase (second four months). Patients take their medicines at home (with or without observation by a treatment supporter) and are expected to return to clinic bi-monthly (intensive phase) or monthly (continuation phase) to check on side effects and obtain refills. At each refill visit, health center staff also assess adherence via patient self-report. Health center staff are supposed to call or visit patients who do not return for refills.

### ***Intervention strategy***

The 99DOTS-based strategy seeks to facilitate TB treatment completion by addressing barriers to traditional facility- or community-based DOT treatment. Importantly, it provides a higher level of treatment supervision than what occurs in routine care. As further described below, it includes the following core components:

| **Component** | | **Barrier addressed** |
| --- | --- | --- |
| 1 | Daily dosing confirmation via toll-free phone calls | Addresses high cost of clinic visits for patients, lack of real-time information for providers on patient adherence to medications. |
| 2 | Daily dosing reminders via automated SMS or pre-recorded phone calls | Addresses high cost of clinic visits for patients and assists with memory and planning processes known to be importance to adherence. |
| 3 | Weekly check-in via automated two-way SMS or interactive voice response phone calls | Addresses lack of social support and feeling of isolation during TB treatment; shown to be effective in other contexts at increasing connection with CHW and reducing social isolation. |
| 4 | Differential management protocol* | Addresses limited time and resources among DTU staff and the need to focus on non-adherent patients. |

* Per current practice, patients will be asked to return to clinic every 2 weeks for refills during the first 4 weeks of treatment. Subsequently, patients deemed to have taken >90% of doses will be asked at the 4- and 8-week visits if they prefer to return to clinic monthly or every 2 months, respectively. In contrast, patients who miss >2 consecutive doses, take <90% of doses overall, or do not respond to the weekly check-in will have enhanced follow-up though phone calls and, if no response, a home visit from a CHW. DTU staff will identify patients who require enhanced follow-up, as well as those eligible for more a more liberalized refill schedule, through the 99DOTS mobile phone app (and document whether follow-up was completed via the app).

### **Randomization**

Following an initial baseline month, the intervention strategy will be implemented at 3 DTUs per month over 6 consecutive months. 18 DTUs will be randomly assigned into six groups (3 DTUs per group), with each group switching from routine care to 99DOTS-based strategy in a randomly assigned order. The order will be unveiled to health center/hospital directors (or their designated representative) at a meeting chaired by the Uganda NTLP Director. Patients initiating TB treatment during the month a site switches to the 99DOTS-based intervention will be excluded from the analysis to allow time for 99DOTS implementation.

**Table 2. 99DOTS Randomization and Enrollment** Schedule

|  | **Month** | | | | | | | |
| --- | --- | --- | --- | --- | --- | --- | --- | --- |
|  | 1 | 2 | 3 | 4 | 5 | 6 | 7 | 8 |
| Group X |  |  |  |  |  |  |  |  |
| Group X |  |  |  |  |  |  |  |  |
| Group X |  |  |  |  |  |  |  |  |
| Group X |  |  |  |  |  |  |  |  |
| Group X |  |  |  |  |  |  |  |  |
| Group X |  |  |  |  |  |  |  |  |

Routine care

Switch to 99DOTS

99DOTS implementation

## Procedures

### ***Pre-implementation site visit***

Project staff will photograph each page of the Unit TB Treatment Register from January 2017 until the start of the study. Project staff will also train two hospital/health center staff (one primary, one backup) identified by the hospital/health center director to take photos of 1) the Unit TB Treatment Register every 2-4 weeks and 2) patient TB Treatment Cards at all refill visits for the duration of the project using a camera-enabled smartphone, and to upload the photos to a central secure server through REDCap mobile. Health center staff will be trained to delete photos from the phone after upload confirmation. Completeness of TB treatment registers will be assessed, and re-training provided as needed.

### ***Health center intervention strategy training***

99DOTS training will occur prior to implementation of the intervention strategy. Project staff and the District TB Officer will conduct the training jointly using standardized training materials. DTU staff will be trained on how to register patients on the 99DOTS platform via a smartphone app, counsel patients regarding use of 99DOTS, use the 99DOTS application to review dosing history and conduct differential management based on dosing history and response to weekly check-ins. We will train CHWs to conduct home visits when needed based on dosing history or response to weekly check-ins.

After the training, DTU staff will be requested to offer 99DOTS-based treatment supervision to all adults initiating TB treatment who have access to a cell phone, and to register patients who accept 99DOTS-based treatment supervision on the 99DOTS platform. The registration process involves entering the same information entered into the routine TB Treatment Register for each patient initiating TB treatment, including patient name, age, gender, address, phone number(s), and treatment start and end dates. The decision to offer and accept 99DOTS-based treatment supervision will be made by DTU staff and patients, respectively.

### ***Patient-level data collection***

Patient demographic and clinical information, dosing information (start and stop date for treatment, monthly number of doses, monthly weight), and treatment outcome data will be extracted from photos of the TB Treatment Registers, TB Treatment Cards and/or the 99DOTS server using standardized data extraction forms (see REDCap Patient Data Extraction Form and 99DOTS Data Capture Tool). Study staff will resolve queries related to missing data in the primary data sources during quarterly site visits.

### ***Process metric data collection***

During the implementation period at each site, process metric data to assess the implementation of each strategy component will be extracted mainly from the 99DOTS server. The server will log all calls made by patients to confirm dosing, all SMS (or IVR phone calls) sent to and received from patients, and documentation of DTU staff/CHW phone calls and home visits. In addition, to confirm SMS were actually received by patients on their handset, DTU staff will request patients not to delete any messages received from 99DOTS until their next refill visit. At each refill visit, DTU staff will request patients to show them any SMS received from 99DOTS since the previous visit and record the number of SMS received.

### ***Patient and provider interviews to refine design of 99DOTS***

In Months 3 and 5, study staff will conduct unstructured interviews with patients and providers at sites that have already implemented the 99DOTS-based intervention strategy. The interviews will be unstructured to elicit participants’ honest and open feedback about their experience using 99DOTS (see DOT to DAT DESIGN: Patient Interview Guide, which contains examples of questions that may be asked to participants). We may seek to record select interviews in order to ensure that we are capturing in-depth insights into users' needs. Audio recordings of interviews will not include any identifiable information. No names or other personal health information will be included in the recordings. Recordings will be anonymous, and we will always ask interviewees before recording and only record if a participant gives his or her verbal consent. The information gained from the interviews will be utilized to further refine the design of 99DOTS.

### ***Patient and provider interviews to identify factors influencing adoption and implementation of the intervention strategy***

Study staff will conduct in depth semi-structured interviews with 5-10 eligible patients and 5-10 eligible providers per site (90-180 total patients, 90-180 total providers) to understand reasons for variability in adoption and implementation of the 99DOTS-based strategy and at selected sites to assess patient costs associated with 99DOTS relative to standard of care (N=5-10/site at 6-8 sites; 30-80 total). The interview will assess relevant constructs of the Consolidated Framework for Implementation Research (CFIR) (see attached DOT to DAT IMPLEMENTATION: Patient Interview Guide and DOT to DAT IMPLEMENTATION: Provider Interview Guide). The CFIR has been widely used to guide evaluation and implementation research related to implementation deployment. Patient costs will be assessed with a tool based on the WHO handbook for tuberculosis patient cost surveys. Interviews will be conducted by trained and experienced staff, audio-recorded, and professionally transcribed.

***Health system costing***

A mix of approaches will be used to collect and evaluate cost and operational data associated with the 99DOTS-based strategy. Eligible providers (5-10 per site; 90-180 total) will also be interviewed to gain a more complete understanding of the activities and staff members involves in the operations of 99DOTS. Time-and-motion studies of 3-5 identified staff members per site will be carried out at one clinic per strategy implementation wave (a total of six clinics overall, purposively sampled to ensure good representation of clinic volume and geography) every three months. These time-and-motion studies will consist of direct observation of all activities conducted by clinic staff over a two-day period per evaluation, for a total 12 days of observation every three months over the course of the 14-month study period. During these observation periods, the time and resources required to perform all activities related to TB treatment (e.g., medication preparation, contacting patients, observing medication doses, etc.) will be recorded.

Although not human subjects research, overhead costs will be estimated using an ingredients approach, incorporating the cost of supplies, building/space, vehicles, and human resources, and then combined with recurrent costs such as building and vehicles maintenance costs, and supplies (such as medical supplies and administrative supplies). We will also estimate the costs of implementing the 99DOTS-based strategy by structured interviews of research staff.

## Study Instruments

1. REDCap Patient Data Extraction Form
2. 99DOTS Data Capture Tool
3. DOT to DAT DESIGN: Patient Interview Guide
4. DOT to DAT IMPLEMENTATION: Patient Interview Guide
5. DOT to DAT IMPLEMENTATION: Provider Interview Guide
6. DOT to DAT Time and Motion Activity Log
7. Generic WHO Patient Costing Survey

## Outcomes

### ***Definitions***

- ***Number treated***: Number of eligible patients identified over a defined 8-month enrollment period through review of the NTLP Treatment register at each study site (includes patients treated for TB without undergoing any sputum testing). Patients started on treatment within 7 days of the 99DOTS strategy training at each site will not be counted.
- ***Date treatment started***: Treatment start date recorded in the NTLP Treatment register and/or 99DOTS server. If discrepant, the NTLP Treatment register will be considered the primary source.
- ***Number enrolled on 99DOTS****:* Number of eligible patients entered into the 99DOTS registration system beginning on the 8^th^ day after the introduction of 99DOTS at the DTU through the end of the 8-month enrollment period.
- ***Date registered on 99DOTS***: Date of registration in 99DOTS as recorded in the 99DOTS server.
- ***Number treated successfully***: Number treated and with a treatment outcome of cured or completed entered into the Unit Treatment register, District Treatment Register, or Unit Treatment Register where patient was transferred.
- ***Number lost to follow***-***up***: Number treated and with a treatment outcome of lost to follow-up entered into the Unit Treatment register OR Unit Treatment Register where patient was transferred AND not recorded as having completed treatment or died in District TB Register.
- ***Number persisting on treatment***: Number treated and documented as having completed at least 60 doses of treatment.
- ***Number converted****:* Number of smear-positive patients started on treatment with documented negative sputum smear result within 2-3 months of date treatment started.

### ***I. Primary Outcome (Effectiveness)***

| **Outcome** | **Numerator** | **Denominator** |
| --- | --- | --- |
| Proportion treated successfully | Number treated successfully | Number treated |

### ***II. Secondary Outcomes (Effectiveness and Cost-Effectiveness***)

| **Outcome** | **Numerator** | **Denominator** |
| --- | --- | --- |
| Proportion with persistence | Number persisting on treatment | Number treated |
| Proportion converted | Number converted | Number treated AND sputum smear-positive pre-treatment |
| Proportion lost to follow-up | Number lost to follow-up | Number treated |
| Incremental cost per patient treated successfully | Incremental cost | Incremental number of patients successfully treated |

###

### ***III. Implementation Outcomes***

| **Implementation period only** | | |
| --- | --- | --- |
| **Outcome** | **Numerator** | **Denominator** |
| Proportion enrolled on 99DOTS | Number enrolled on 99DOTS | Number treated |
| Proportion of scheduled doses confirmed by phone call | Number of scheduled doses confirmed by phone call | Number enrolled on 99DOTS (or number called?) |
| Proportion of daily SMS sent by 99DOTS platform | Number of daily SMS sent by 99DOTS | Number enrolled on 99DOTS |
| Proportion of daily SMS received on patient handset | Number of daily SMS received on patient handset | Number of daily SMS sent by 99DOTS |
| Proportion of weekly SMS sent by 99DOTS platform | Number of weekly SMS sent by 99DOTS | Number enrolled on 99DOTS |
| Proportion of weekly SMS received on patient handset | Number of weekly SMS received on patient handset | Number of weekly SMS sent by 99DOTS |
| Proportion of weekly SMS to which patients send a response | Number of weekly SMS to which patients send a response | Number of weekly SMS received on patient handset |
| Proportion of patients followed-up via phone call or home visit if responding No or not responding to weekly check-in | Number of patients follow-up via phone call or home visit | Number of patients responding No to weekly check-in |
| Proportion of patients called or visited by DTU staff/CHW when missing >2 consecutive doses | Number of patients called or visited by DTU staff/CHW | Number of patients missing >2 consecutive doses |

Source of data: ^1^ Treatment Register; ^2^ Treatment Card; ^3^ 99DOTS server

## Data Management

Dr. Phillips (statistician) will oversee data management in conjunction with UCSF- and Uganda-based study coordinators using the NIH-recommended Research Electronic Data Capture (**REDCap**) software, password-protected and accessible only to research staff. All data will be entered into standardized REDCap forms, with validation of data using range and consistency checks. Quality control procedures will include review of all study data sources for completeness and accuracy prior to data capture. Study staff will begin data capture by compiling photos from each individual health center for each data collection period. Using patient name, age, and sex, the staff will create one unique REDCap database entry (*i.e.* record) per patient by tracking patients across data sources in the following order: 1) Unit TB Treatment Register; 2) TB Treatment Card; 3) 99DOTS server (implementation period only). Once patients have been matched across all data sources, the staff will run a series of reports in REDCap to verify the completeness and accuracy of key variables impacting patient eligibility and/or study outcomes: age, sex, HIV status, smear status, outcomes dates (start and end of treatment, treatment outcome). Study staff will review the original data photographs to verify the information is missing, and they will compile a list of follow up items for each health center. Research staff will phone health center staff after each 2- to 4-week period for which data is extracted to resolve missing information and clarify any discrepancies or uncertainties in matching. Once the health center reviews the missing and/or inaccurate data, the study staff will update REDCap. The UCSF study coordinator will visit Uganda 2-3 times a year and review a random sample of forms and primary data sources for quality assurance.

## Statistical Analysis

### ***Aim 1***

A detailed Statistical Analysis Plan will be developed. Briefly, we will calculate and compare all effectiveness outcomes for the two study arms using an intention-to-treat analysis. Mixed effects modeling analytical methods common for stepped-wedge designs^8^ will be used to assess the effect of the strategy on effectiveness outcomes, incorporating both fixed (*i.e.,* strategy and time) and random (*i.e*., cluster) factors. These methods will consider time as a potential confounder due to the design of SWTs where the strategy is gradually introduced to a few clusters at a time over the course of the enrollment period, resulting in the control arm contributing data from an earlier point in time than the strategy arm. The primary outcome definitions will be recorded as binary (patient treated successfully = 1; patient not treated successfully = 0), with the proportion of patients treated successfully anticipated to increase with the strategy. Risk difference and relative risk will be reported along with 95% confidence intervals.

### ***Aim 2***

*1) Descriptive analyses* – We will report process metrics on a monthly basis to assess adoption and maintenance of each strategy component overall, within key patient sub-groups, and at individual sites.

*2) Comparative analyses* – To identify patient-, provider-, and/or clinic-level factors independently associated with adoption and maintenance of strategy components, we will develop linear or logistic regression models, taking into account the clustered design (for example, robust standard errors).

### ***Aim 3***

*Cost-effectiveness --*Our primary outcome will be the incremental cost-effectiveness of the intervention strategy from a societal perspective, measured as the incremental cost per successfully completed treatment, comparing 99DOTS relative to the standard-of-care of community based DOTS treatment. To assess cost-effectiveness, we will estimate the incremental cost of 99DOTS using an “ingredients” (bottom-up) approach, thus incorporating costs of all individual components including human resources, consumables, implementation, overheads, and any capital costs (e.g., building space, equipment) needed. Incremental patient costs will also be assessed. We will estimate the incremental effectiveness of 99DOTS as described in Aim 1 above. Our primary outcome measure will be the ratio of incremental costs to incremental effectiveness (incremental cost-effectiveness ratio, or ICER). In secondary analyses we will consider the healthcare perspective (as opposed to the societal perspective) and will also construct a Markov model of clinical outcomes to convert incremental effectiveness (number of successfully completed treatments) into estimates of deaths and DALYs averted. Data to inform transition probabilities and health utilities in this model will come largely from the literature. We will follow international conventions for all procedures including economic costing, discounting, and reporting. We will conduct one-way sensitivity analyses across all model parameters, multi-way sensitivity analyses for those parameters found to be most influential, and a probabilistic uncertainty analysis in which all parameters are varied simultaneously using Latin Hypercube Sampling.

## Sample Size Considerations

### ***Aim 1***

The study aims to demonstrate the superiority of the 99DOTS-based strategy. The sample size calculation uses formulae appropriate for stepped-wedge evaluations.^9^ The primary outcome is the proportion of patients treated successfully. A type I error of 5% and power of at least 90% is assumed. Based on 2017 data, the harmonic mean number of patients initiating treatment for drug-susceptible PTB per month across project DTUs is 15. Thus, we anticipate approximately 1890 patients will initiate treatment over the 8-month enrollment period (945 in the pre- and 945 in the post-implementation phases across DTUs). We will have 89% power to demonstrate that our strategy increases the proportion of patients treated successfully by 10% or more (assumptions: alpha=0.05; ICC = 0.001 calculated using 2017 NTLP data for the 18 DTUs; pre-implementation treatment success = 51% based on 2017 NTLP data for the 18 DTUs; calculations performed using *steppedwedge* command in Stata 14).

### ***Aim 2***

The sample size for Aim 2 analyses is fixed by parameters of the overall study. For quantitative analyses, the sample size is sufficiently large (945 patients) to enable multivariable analysis to identify factors associated with intervention strategy adoption and maintenance.

### ***Aim 3***

Sample size considerations for the cost data (i.e., number of direct observations through time-and-motion studies, number of patients completing the cost interviews) are based on feasibility considerations and desire to estimate model parameters to sufficient levels of precision, as described above for Aim 2. The sample size for the incremental effectiveness component is described above for Aim 1.

# Ethical Considerations

## Potential risks to participants

There are minimal risks to participants in this study. The primary risk to patients treated for TB during the study period is the potential for loss of confidentiality and stigma should their personal health information, including HIV or TB status, be disclosed. Of note, patients enrolled on 99DOTS will have a greater level of treatment monitoring that is afforded in routine care (as ingestion of each dose is monitored). Participants who are health care providers may be at minimal risk for inconvenience due to time for participation and possible repercussions of answering sensitive questions about their work and workplace. Furthermore, health system provider participants for time-and-motion data collection may feel that they are being individually evaluated for the work they perform for the time-and-motion data collection.

## Protection against risk

The study will be submitted for approval to the Research Ethics Committees of the University of California San Francisco and Makerere University School of Public Health, and to the Uganda National Council for Science and Technology. All study staff will be required to have completed Human Subjects Research Training.

During the implementation period at each health center, the decision to use routine vs. 99DOTS-based treatment supervision will be made by patients and their providers. To minimize the potential for loss of confidentiality, all study data will be stored in locked or password-protected cabinets or databases accessible only to study personnel. Patient names will be used to match patient records across data sources, but will not be included in the password-protected, electronic study database. Photos of TB Treatment Registers and Treatment Cards will be destroyed once entry of data into the electronic study database is completed.

Individual patients evaluated for TB at participating health centers will not be consented because the study meets the requirements to qualify for waiver of informed consent under U.S. Department of Health and Human Services (DHHS) Regulation 46.116 (d): 1) No data or samples will be collected specifically for research purposes; 2) Patients will receive the same or higher quality of care; and 3) It is not practical for health workers to obtain informed consent during the process of delivering routine clinical care.

We will obtain verbal consent from patients and clinic providers participating in the interviews and time-and-motion analyses. A member of the research team will read the verbal consent script to each participant and answer any questions. They will emphasize that participation is voluntary, that consent can be withdrawn at any time, and that a participant can refuse to answer any specific question. The verbal consent process will be audio recorded. Written consent will not be obtained because no participant identifiers will be collected, and the consent document would be the only record linking the participant to the study.

### ***Data protection***

Study data will be entered into a REDCap database, which is hosted on a secure server at UCSF. All study staff will have a unique username and password. No patient identifiers will be exported as part of analysis datasets.

Patient data entered into 99DOTS is stored on Everwell Health Solution’s Microsoft Azure server, which provides best-in-class reliability and physical security. Access to server data via the 99DOTS app or website for DTU staff and NTLP supervisors at participating health centers will be restricted by username and password.  User-level access control for study staff and investigators will be on a need-to-know basis and discontinued once the study is over.

## Potential benefits of the study to participants and society

Patients treated during the implementation period may benefit from greater convenience and reduced cost. They may also benefit from enhanced monitoring of treatment adherence and follow-up if non-adherent to medications relative to what is done in routine care.

Potential benefits to society include identification of strategies to improve TB treatment completion and thereby reduce transmission and amplification of drug resistance. If successful, the proposed strategy could potentially be scaled up to improve TB care in similar settings.

## Dissemination

The study results will be communicated to stakeholders through dissemination meetings and to participating health centers using language-appropriate information sheets. Investigators will present results at relevant conferences and submit manuscript(s) to peer-reviewed journals. Public access to the participant-level dataset of main study results and statistical code will be made available.

## Study governance

Because of the low-risk nature of the research, the Principal Investigator will be responsible for monitoring the data, assuring protocol compliance, and conducting safety reviews on a quarterly basis. External monitoring will be provided by a TB REACH project officer (Amera Khan) and Monitoring and Evaluation consultant (Farihah Malik). The Principal Investigator will submit regular progress reports, including recommendations on whether the project should continue unchanged, require modification/amendment, or close to enrollment. All major modifications (e.g., study design, sample size, study termination or suspension) will be approved by the TB REACH project officer and ethics committees.

# Abbreviations

ART Anti-retroviral therapy

CFIR Consolidated Framework for Implementation Research

CHW Community Health Worker

CRT Cluster-randomized trial

DAT Digital Adherence Technology

DHHS Department of Health & Human Services

DOT(S) Directly-Observed Therapy

DTU Designated Treatment Unit

GCP Good Clinical Practice

ICER Incremental Cost-Effectiveness Ratio

ICH International Council for Harmonisation of Technical

Requirements for Pharmaceuticals for Human Use

IRB Institutional review board

IVR Interactive Voice Response

NIH National Institutes of Health

NTLP National Tuberculosis and Leprosy Program

NTP National TB Program

PRECEDE Predisposing, Reinforcing, and Enabling Constructs in Educational/Environmental Diagnosis and Evaluation

PTB Pulmonary Tuberculosis

REDCap Research Electronic Data Capture

SMS Short Messaging Service

TB Tuberculosis

UCSF University of California San Francisco

WHO World Health Organization

# Competing interests

The investigators declare that they have no competing interests.

# References
