# Supplementary material for: Digital adherence technology for tuberculosis treatment supervision: A stepped-wedge cluster-randomized trial in Uganda
Source: PLoS Med. 2021 May 6;18(5):e1003628. doi: 10.1371/journal.pmed.1003628 (PMC8136841; doi:10.1371/journal.pmed.1003628)
Supplement: Attachment — Submitted filename: 99DOTS Uganda trial - PLOS Med 2021 review.docx [file pmed.1003628.s010.docx]

This is an important and well-conducted stepped-wedge cluster randomized trial assessing the impact of implementing a digital adherence technology (DAT) on TB treatment outcomes in a low-income country. With ongoing critique of DOT models in TB care, interest in DATs—as a replacement for DOT—has been growing; however, there is a paucity of evidence regarding the impact of these technologies on TB treatment outcomes and longer-term outcomes, particularly in low- and middle-income country settings. As such, this study is a potentially important contribution.

While the study is well-conducted, I have considerable concerns about the interpretation and framing of the study findings, particularly with regard to the per protocol analysis, which seems to drive the entire Discussion section of the paper. While the authors appropriately interpret the findings of the intent to treat analysis—that 99DOTS-based treatment supervision did not improve overall treatment outcomes in the patient population—the very substantial limitations of the per protocol analysis raise questions about their interpretation that “99DOTS improved treatment outcomes among patients who received the intervention.” I worry that, given the considerable limitations of the per protocol analysis, this finding could be very misleading, especially given the lack of transparent discussion of these limitations—which I believe are significant enough that they should be emphasized in the abstract, author summary, and discussion sections, so as to signal to the reader that the study findings have not ultimately answered the question of whether this intervention improves outcomes. I outline the reasons for my concerns further below.

Major feedback:

**1. Interpretation of the per protocol analysis findings:** Per protocol analyses have the potential to lead to erroneous findings of intervention effect because—particularly when the analysis excludes a large number of patients that were in the intent to treat analysis—it breaks the random assignment that a randomized trial is supposed to achieve. As such, the correct interpretation of these findings is not necessarily that outcomes improved only among those who received the intervention, but rather that—given the exclusion of a large number of the original patients included in the trial—it remains unclear whether the intervention caused the improved outcomes, or whether the patients left behind in the per protocol analysis are those who were likely to have better outcomes anyways even without the intervention (i.e., introduction of selection bias). In the Discussion section, the authors allude very briefly to this limitation of their study before minimizing it when they state: “It is possible that the patients who were enrolled on 99OTS would have been more likely to complete the treatment even under routine care.”

I worry that this is a much bigger problem than they suggest, for the following reasons:

(a) Per protocol findings may be more likely to reveal a real effect if there is at least some concordance in the directionality of the finding in the ITT analysis (even if the ITT findings were not significant). However, the ITT findings here don’t have the same direction (the odds ratio nicely straddles 1) as the PP analysis findings.
(b) PP findings may be more likely to reveal real effect if it excludes a small proportion of patients; however, this PP analysis excluded nearly half (48%) of study participant in the intervention arm, while only about 3% were excluded from the control group, which could introduce a high level of bias into the findings, as the PP intervention group essentially may represent a non-random sample of the intervention group.
(c) Further the discretion exercised by both healthcare workers and patients in deciding which patients should be enrolled in 99DOTS (from the healthcare workers’ side) and whether to engage with 99DOTS (from the patients’ side) suggest a possibility that patients who enrolled in 99DOTS may have had higher baseline interest in engaging in care and may have been likely to have better outcomes even without the intervention. As such, when the PP analysis restricts to this subset of patients in the intervention group, it is hard to know whether the effect seen is a result of the intervention or of selection bias in the PP analysis. This is of course not the fault of the authors or the study design – but it does speak to the importance of placing precedence on the findings from the ITT analysis (which showed no effect), and of interpreting the PP analysis cautiously, rather than suggesting it shows a clear treatment effect. It’s not clear if the PP analysis has much validity given the loss of half of the intervention sample.
(d) In addition, 6.5% of those who didn’t enroll in 99DOTS died or were lost to follow-up before they could enroll and another 4.2% were deemed too ill or elderly to enroll – presumably they weren’t included in the PP intervention group. The 6.5% all clearly had bad outcomes while the 4.2% were highly likely to have bad outcomes – and, from what I can tell, early deaths or severely ill individuals were not excluded from the PP control arm. This is a very clear source of bias in favor of seeing a positive effect from the 99DOTS/intervention group. In fact, just the exclusion of this ~10% of patients from the intervention period group could account for much of the ~10% difference in outcomes seen between the control and intervention periods in the PP analysis. Again, highlights the concerns about the PP analysis and the importance of highlighting these limitations and prioritizing the finding from the ITT analysis.
(e) Finally, the authors do a post-hoc analysis in which they compare the individuals in the intervention period/group in the PP analysis to those in the control/period group who had phone numbers listed on their treatment cards, in a reasonable attempt to control for the bias in the PP intervention group. However, their description of patients who engaged with 99DOTS suggests that it may not match well with this subset of patients in the control period. For example, 65% of patients who didn’t enroll in 99DOTS in the intervention period didn’t have regular access to a phone – which suggests one-third of patients actually did have regular access to a phone but still chose not to enroll in 99DOTS, again raising concerns about those who enrolled with 99DOTS being more engaged with their TB care even without the intervention. As such the intervention period/group in the PP analysis excludes many patients who actually had regular access phones. Also, in the modified PP control group, just having a phone number listed on the treatment card does not mean that these patients will have regular access to their phones – which is another reason to why this modified PP control group probably does not actually address the problem of bias in the PP analysis.

**2. Importance of IIT findings versus PP**: Everything described in major critique #1 above does not undermine the trial study design per se – it just highlights that the primary valid finding is the finding from the ITT analysis, and the one from the PP is interesting but at such high risk of bias that using it as the focus of the paper could be misleading in suggesting 99DOTS has a treatment effect when it may or may not. The framing is critical. If the discussion is framed around the ITT findings, the reader walks away viewing this as an intervention with no clear benefit to the overall population of TB patients, but that may still be worth investigating further. Such a framing, which is more appropriate in my mind, supports further research rather than a “scaling up” of 99DOTS as advocated by the authors. The current discussion framed around the PP findings has readers walking away thinking that this intervention has clear benefit to those who use it, when this is far from clear given the substantial limitations and bias in the PP analysis – and therefore the current discussion is misleading. The ITT findings are clearly the scientifically sound findings.

In addition to reframing the discussion, a robust discussion of the limitations and biases in the PP analysis should be included in the Discussion section.

**Minor feedback:**

1. On page 5, the authors state that “The most common DAT platform…within an electronic pillbox.” Electronic pillboxes certainly are being used at scale in China, but there are many other DATs in wide use – including video DOT in high-income settings. In fact, the most commonly used DAT is possibly 99DOTS, given its very large-scale rollout to hundreds of thousands of patients in India, Myanmar, and elsewhere. As such, revise this to say “A common DAT platform…”
2. On page 18, “To our knowledge, these data represent the first to show the impact of a low-cost DAT on TB treatment outcomes.” – This isn’t a correct statement, as the authors note in the very same paragraph. The Yoeli et al. trial of a two-way SMS platform in Kenya that the authors discussed showed an decline in unfavorable treatment outcomes (and therefore, conversely, an improvement in favorable outcomes). Also, if the authors take major critique #1 above seriously, then this also would not be a true statement b/c it is not clear that this study shows a real impact on treatment outcomes, if they prioritize the ITT findings.
3. There is some observational literature on TB treatment outcomes with 99DOTS in India – see Thekkur et al. Global Health Action 2019;12(1):1568826. The study finds poorer treatment outcomes at sites that rolled out 99DOTS, even after some adjustment for differences in patient characteristics. This is of course an observational study with its own risk of bias – however, there is similar risk of bias in the current PP analysis given the large proportion of patients excluded in the intervention period. It would be helpful for the authors to discuss their study findings in the context of this prior research on 99DOTS. There is also research on 99DOTS accuracy for measuring adherence and patient engagement with the technology that might be helpful to contextualize their findings.
