# Supplementary material for: Digital adherence technology for tuberculosis treatment supervision: A stepped-wedge cluster-randomized trial in Uganda
Source: PLoS Med. 2021 May 6;18(5):e1003628. doi: 10.1371/journal.pmed.1003628 (PMC8136841; doi:10.1371/journal.pmed.1003628)
Supplement: Attachment — Submitted filename: PLOS Med Response to Reviewers_2021.2.24.docx [file pmed.1003628.s011.docx]

**Response to reviewers**

**PLOS Medicine Manuscript Number PMEDICINE-D-20-06186R1**

Dear Editorial Board,

Thank you for the opportunity to submit a revised draft of our manuscript entitled “Digital adherence technology for tuberculosis treatment supervision: a stepped-wedge cluster randomized trial.” We appreciate the reviewers’ detailed comments. They have helped us to substantially improve the manuscript. Please see below, in blue, our responses to the reviewer’s comments.

**Requests from the editors:**

Please add a new final sentence to the "Methods and findings" subsection of your abstract, which should begin "Study limitations include ..." or similar and quote 2-3 of the study's main limitations.

**Author Response:** We added the sentence, “Study limitations include the likelihood of selection bias in the PP analysis, inability to verify medication dosing in either arm and incomplete implementation of some components of the intervention.”

Do you mean "waiver of informed consent" in the Methods section?

**Author Response:** Yes, this has been corrected to read, “A waiver of informed consent was granted to access patient demographic and clinical information recorded in TB treatment registers.”

Please remove trademarks, e.g., in the Methods section. 

**Author Response:** We have removed the trademark after Stata in the Methods section. It now reads, “Stata 15 was used for all analyses.”

Throughout the text, please ensure that reference call-outs fall before punctuation, e.g., "... blister packs [5,6]." (noting the absence of spaces in the square brackets). 

**Author Response:** We have moved all references to fall before punctuation and removed spaces within square brackets.

Please remove the information on study funding from the "Acknowledgments" section at the end of the main text. Funding information should appear in the article metadata, via entries in the submission form. 

**Author Response:** This has been removed.

In the reference list, please ensure that journal names are abbreviated consistently, e.g., "PLoS Med.".

**Author Response:** We have made sure all journal names are abbreviated consistently.

Are references 4 & 18 duplicates?

**Author Response:** Yes. We have removed reference 18.

Please update reference 8. 

**Author Response:** We have updated reference 8 to reflect the published version:

Crowder R, Kityamuwesi A, Kiwanuka N, Lamunu M, Namale C, Tinka LK, et al. Study protocol and implementation details for a pragmatic, stepped-wedge cluster randomised trial of a digital adherence technology to facilitate tuberculosis treatment completion. BMJ Open. 2020;10(11):e039895.

Please spell out the institutional author name for reference 13.

**Author Response:** We removed this reference in the revised submission.

Please adapt the attached CONSORT checklist so that individual items are referred to by section (e.g., "Methods") and paragraph number rather than by line or page numbers, as the latter generally change in the event of publication. 

**Author Response:** We have updated the CONSORT checklist to refer to individual items by manuscript section and paragraph number.

Please name the checklist "S1_CONSORT_Checklist" or similar, and refer to it by this label in your Methods section. Please also refer to the attached trial protocol document in the same way.

**Author Response:** We have referenced the trial protocol, statistical analysis plan, and CONSORT Checklist at the end of the first sub-section of the Methods and re-numbered other supplementary materials accordingly: “The trial protocol has been published previously [8], and copies of the trial protocol, statistical analysis plan, and CONSORT checklist are available as supporting information (S1 Trial Protocol, S1 Statistical Analysis Plan, S1 CONSORT Checklist).”

**Comments from the reviewers:**

*****Reviewer #1:** 

This is an important and well-conducted stepped-wedge cluster randomized trial assessing the impact of implementing a digital adherence technology (DAT) on TB treatment outcomes in a low-income country. With ongoing critique of DOT models in TB care, interest in DATs—as a replacement for DOT—has been growing; however, there is a paucity of evidence regarding the impact of these technologies on TB treatment outcomes and longer-term outcomes, particularly in low- and middle-income country settings. As such, this study is a potentially important contribution.

While the study is well-conducted, I have considerable concerns about the interpretation and framing of the study findings, particularly with regard to the per protocol analysis, which seems to drive the entire Discussion section of the paper. While the authors appropriately interpret the findings of the intent to treat analysis—that 99DOTS-based treatment supervision did not improve overall treatment outcomes in the patient population—the very substantial limitations of the per protocol analysis raise questions about their interpretation that "99DOTS improved treatment outcomes among patients who received the intervention." I worry that, given the considerable limitations of the per protocol analysis, this finding could be very misleading, especially given the lack of transparent discussion of these limitations—which I believe are significant enough that they should be emphasized in the abstract, author summary, and discussion sections, so as to signal to the reader that
the study findings have not ultimately answered the question of whether this intervention improves outcomes. I outline the reasons for my concerns further below.

Major feedback:

1. Interpretation of the per protocol analysis findings: Per protocol analyses have the potential to lead to erroneous findings of intervention effect because—particularly when the analysis excludes a large number of patients that were in the intent to treat analysis—it breaks the random assignment that a randomized trial is supposed to achieve. As such, the correct interpretation of these findings is not necessarily that outcomes improved only among those who received the intervention, but rather that—given the exclusion of a large number of the original patients included in the trial—it remains unclear whether the intervention caused the improved outcomes, or whether the patients left behind in the per protocol analysis are those who were likely to have better outcomes anyways even without the intervention (i.e., introduction of selection bias). In the Discussion section, the authors allude very briefly to this limitation of their study before minimizing it when they
state: "It is possible that the patients who were enrolled on 99OTS would have been more likely to complete the treatment even under routine care."

I worry that this is a much bigger problem than they suggest, for the following reasons:

(a)    Per protocol findings may be more likely to reveal a real effect if there is at least some concordance in the directionality of the finding in the ITT analysis (even if the ITT findings were not significant). However, the ITT findings here don't have the same direction (the odds ratio nicely straddles 1) as the PP analysis findings.
(b)    PP findings may be more likely to reveal real effect if it excludes a small proportion of patients; however, this PP analysis excluded nearly half (48%) of study participant in the intervention arm, while only about 3% were excluded from the control group, which could introduce a high level of bias into the findings, as the PP intervention group essentially may represent a non-random sample of the intervention group.
(c)    Further the discretion exercised by both healthcare workers and patients in deciding which patients should be enrolled in 99DOTS (from the healthcare workers' side) and whether to engage with 99DOTS (from the patients' side) suggest a possibility that patients who enrolled in 99DOTS may have had higher baseline interest in engaging in care and may have been likely to have better outcomes even without the intervention. As such, when the PP analysis restricts to this subset of patients in the intervention group, it is hard to know whether the effect seen is a result of the intervention or of selection bias in the PP analysis. This is of course not the fault of the authors or the study design - but it does speak to the importance of placing precedence on the findings from the ITT analysis (which showed no effect), and of interpreting the PP analysis cautiously, rather than suggesting it shows a clear treatment effect. It's not clear if the PP analysis has much validity
given the loss of half of the intervention sample.
(d)    In addition, 6.5% of those who didn't enroll in 99DOTS died or were lost to follow-up before they could enroll and another 4.2% were deemed too ill or elderly to enroll - presumably they weren't included in the PP intervention group. The 6.5% all clearly had bad outcomes while the 4.2% were highly likely to have bad outcomes - and, from what I can tell, early deaths or severely ill individuals were not excluded from the PP control arm. This is a very clear source of bias in favor of seeing a positive effect from the 99DOTS/intervention group. In fact, just the exclusion of this ~10% of patients from the intervention period group could account for much of the ~10% difference in outcomes seen between the control and intervention periods in the PP analysis. Again, highlights the concerns about the PP analysis and the importance of highlighting these limitations and prioritizing the finding from the ITT analysis.
(e)    Finally, the authors do a post-hoc analysis in which they compare the individuals in the intervention period/group in the PP analysis to those in the control/period group who had phone numbers listed on their treatment cards, in a reasonable attempt to control for the bias in the PP intervention group. However, their description of patients who engaged with 99DOTS suggests that it may not match well with this subset of patients in the control period. For example, 65% of patients who didn't enroll in 99DOTS in the intervention period didn't have regular access to a phone - which suggests one-third of patients actually did have regular access to a phone but still chose not to enroll in 99DOTS, again raising concerns about those who enrolled with 99DOTS being more engaged with their TB care even without the intervention. As such the intervention period/group in the PP analysis excludes many patients who actually had regular access phones. Also, in the modified PP control
group, just having a phone number listed on the treatment card does not mean that these patients will have regular access to their phones - which is another reason to why this modified PP control group probably does not actually address the problem of bias in the PP analysis.

2. Importance of IIT findings versus PP: Everything described in major critique #1 above does not undermine the trial study design per se - it just highlights that the primary valid finding is the finding from the ITT analysis, and the one from the PP is interesting but at such high risk of bias that using it as the focus of the paper could be misleading in suggesting 99DOTS has a treatment effect when it may or may not. The framing is critical. If the discussion is framed around the ITT findings, the reader walks away viewing this as an intervention with no clear benefit to the overall population of TB patients, but that may still be worth investigating further. Such a framing, which is more appropriate in my mind, supports further research rather than a "scaling up" of 99DOTS as advocated by the authors. The current discussion framed around the PP findings has readers walking away thinking that this intervention has clear benefit to those who use it, when this is far from
clear given the substantial limitations and bias in the PP analysis - and therefore the current discussion is misleading. The ITT findings are clearly the scientifically sound findings.

In addition to reframing the discussion, a robust discussion of the limitations and biases in the PP analysis should be included in the Discussion section.

**Author Response (Comments 1 and 2):**  We thank the reviewer for these extensive and well considered comments. We largely agree with the stated limitations of the per protocol analysis and the recommendation to re-frame the Discussion. We also agree we should not claim that the study showed that 99DOTS-based treatment supervision improved treatment outcomes and have removed such text wherever it appeared. Even though 99DOTS-based treatment supervision did not improve treatment outcomes in the population as a whole, we believe the findings are consistent with it being a viable alternative to DOT for many patients as also pointed out by other reviewers. Specifically, over half of all eligible participants were able to engage in an intervention that did not require direct observation of therapy – and those who did engage in 99DOTS-based treatment supervision achieved good treatment outcomes (including >85% treatment completion).

We have extensively revised our manuscript in an attempt to provide this more nuanced message, while trying to be very clear that 99DOTS-based treatment supervision did not improve treatment outcomes in the population as a whole (i.e., in the ITT analysis). For example, we now conclude that 99DOTS could be offered as an option for patients and their health workers to choose as an alternative to directly observed therapy – but only after clarifying that 99DOTS-based treatment supervision did not improve treatment outcomes in the full population. Below, we highlight some of the specific changes made in response to the comments raised by the Reviewer:

- Abstract conclusion modified to: “99DOTS-based treatment supervision did not improve treatment outcomes in the overall study population. However, similar treatment outcomes were achieved during the control and intervention periods, and those patients enrolled on 99DOTS achieved high treatment completion. 99DOTS-based treatment supervision could be a viable alternative to directly observed therapy for a substantial proportion of patients with TB.”
- Author Summary - What did the researchers do and find?: Replaced text indicating the trial demonstrated improved TB treatment outcomes:
  - “Only about half of patients were initiated on 99DOTS-based treatment supervision during the intervention period.”
  - “The 99DOTS-based intervention did not increase treatment completion in the full study population.”
  - “Treatment completion was high (>85%) among the non-random sample of patients initiated on 99DOTS-based treatment supervision during the intervention period.”
- Author Summary - What do the findings mean?: Replaced text suggesting that 99DOTS should replace DOT:
  - “99DOTS – and likely other digital adherence technologies – should not be used as a universal replacement for directly observed therapy for TB treatment supervision, with the aim of increasing population-level treatment completion.”
  - “99DOTS-based treatment supervision could enable a substantial proportion of patients with TB to complete treatment without the inconvenience and additional costs of directly observed therapy.”
  - “Further research is needed to assess whether overall treatment outcomes can be improved by increasing uptake of 99DOTS or other low-cost DATs, and what additional measures are needed to support all patients to achieve cure regardless of the mode of treatment supervision.”
- Results, Figure 3: We modified Figure 3 (formerly Figure 4) to include the sub-group findings from the ITT analysis to align with the reviewer’s comment to maintain a focus on the ITT analysis.
- Discussion: 1^st^ paragraph now highlights high risk of bias in the per protocol analysis and concludes that 99DOTS should not replace DOT but could be an alternative for many patients:

“In this stepped-wedge cluster-randomized trial of 1,913 patients with drug-susceptible TB in Uganda, there was overall no significant difference in outcomes between the control and intervention periods. Only 52% of patients in the intervention period were enrolled on 99DOTS-based treatment supervision; in the per-protocol analysis, these patients were substantially more likely to complete treatment, including persisting on treatment through the intensive phase and avoiding loss to follow-up during treatment. Thus, while 99DOTS-based treatment supervision does not improve population-level treatment outcomes relative to DOT, it is likely a viable alternative to DOT for the substantial proportion of patients who have access to a phone and are interested to use this technology.”

- Discussion, new 3^rd^ paragraph highlights biases in per protocol analysis as nicely outlined by the reviewer:

“Due to the pragmatic nature of our trial, health workers and patients made all decisions about the use of 99DOTS during the intervention period, and only about half of all eligible patients were enrolled on 99DOTS. The substantially better treatment outcomes among these patients in the per protocol analysis should not be interpreted as improving outcomes in the overall population, primarily due to the possibility of selection bias. Patients enrolled on 99DOTS likely represent a non-random sample of patients initiating treatment with higher interest in engaging in care and therefore higher likelihood of completing treatment even without 99DOTS. Although our post-hoc analysis excluding patients without listed phone numbers showed similar findings to the per protocol analysis, it may not adequately control for this bias – the reason for non-enrollment on 99DOTS was unrelated to phone access for approximately one-third of patients in the intervention period. Despite these caveats, it is noteworthy that 99DOTS-based treatment supervision empowered half of all patients to take TB medicines at the time and place of their choosing, resulting in >85% treatment completion while mitigating the potential inconvenience, stigma and costs commonly reported as barriers associated with DOT [20-22]. Further research is needed on whether overall treatment outcomes can be improved by increasing the uptake of low-cost DATs such as 99DOTS (e.g., by providing patients with phones or offering a choice of different DAT platforms) and strengthening the capacity of health workers to provide enhanced adherence support using real-time dosing information.”

- Discussion, paragraph on limitations now also lists potential for bias in the PP analysis:

“As above, the PP analyses should be interpreted with caution given the probability of selection bias in the PP population.”

- Discussion, last paragraph modified to reflect more nuanced interpretation of findings:

“In conclusion, our trial provides randomized evidence that 99DOTS-based treatment supervision does not improve population-level treatment outcomes. However, the high levels of treatment completion among those who used 99DOTS suggest that this technology may be a viable alternative to DOT for many patients. Given that 99DOTS likely reduces costs and burden to patients, these findings support a more patient-centered approach to TB treatment supervision that replaces universal DOT with offering 99DOTS-based treatment supervision as an alternative treatment supervision modality for those patients who are willing and able to use it.”

Minor feedback:

1.    On page 5, the authors state that "The most common DAT platform…within an electronic pillbox." Electronic pillboxes certainly are being used at scale in China, but there are many other DATs in wide use - including video DOT in high-income settings. In fact, the most commonly used DAT is possibly 99DOTS, given its very large-scale rollout to hundreds of thousands of patients in India, Myanmar, and elsewhere. As such, revise this to say "A common DAT platform…"

**Author response**: We have edited this sentence to read, “A common DAT platform generates real-time adherence data by placing medications within an electronic pill box.”

2.    On page 18, "To our knowledge, these data represent the first to show the impact of a low-cost DAT on TB treatment outcomes." - This isn't a correct statement, as the authors note in the very same paragraph. The Yoeli et al. trial of a two-way SMS platform in Kenya that the authors discussed showed an decline in unfavorable treatment outcomes (and therefore, conversely, an improvement in favorable outcomes). Also, if the authors take major critique #1 above seriously, then this also would not be a true statement b/c it is not clear that this study shows a real impact on treatment outcomes, if they prioritize the ITT findings.

**Author response**: We hope the reviewer agrees that the substantial changes to the manuscript summarized in our response to major critique #s 1 and 2 reflect that we have taken the feedback seriously. We agree that the sentence noted above is not a correct statement and have removed the sentence in the revised Discussion.

3.    There is some observational literature on TB treatment outcomes with 99DOTS in India - see Thekkur et al. Global Health Action 2019;12(1):1568826. The study finds poorer treatment outcomes at sites that rolled out 99DOTS, even after some adjustment for differences in patient characteristics. This is of course an observational study with its own risk of bias - however, there is similar risk of bias in the current PP analysis given the large proportion of patients excluded in the intervention period. It would be helpful for the authors to discuss their study findings in the context of this prior research on 99DOTS. There is also research on 99DOTS accuracy for measuring adherence and patient engagement with the technology that might be helpful to contextualize their findings.

**Author response**: We thank the reviewer for this suggestion and have incorporated a brief discussion of prior studies including the one cited above at the end of the second paragraph of the Discussion:

“Previous observational studies of 99DOTS in India have reported variable acceptance by patients [17], sub-optimal accuracy for measuring adherence [18] and worsening of treatment outcomes following its implementation [19]. Our trial confirms that 99DOTS – and likely other DATs – are unlikely on their own to substantially improve population-level TB treatment outcomes when implemented as part of routine care.”

***** Reviewer #2:** 

Improved tuberculosis (TB) treatment outcomes were observed in a controlled trial of over 2200 participants in Uganda in 2018-2019 when patients contacted their healthcare giver on a freephone service after taking medications, as against in person direct observation of treatment. The article includes novel findings from the study of 99DOTS, a low-tech digital adherence technology that has been implemented at scale in India and elsewhere ahead of completion of trials. There is thus a lot of interest in the finding of this RCT (esp. during lockdowns and also for the delivery of other treatments, like ART). Moreover the adaptations to the basic technology that were introduced by the investigators of the Uganda trial (educational messages to callers and more neutral packaging) appear to be helpful and could be integrated in the basic package of 99DOTS. In my opinion this work can be published subject to some minor revisions to improve understanding and the authors should be
allowed a reasonable excess over the word limit to explain certain key issues of their interesting research and its implications adequately:

page 2, line 1: treatment completion for new & relapse cases is on target globally (at around 85%). Maybe you can state that in many countries (including Uganda) adherence and treatment completion remain problematic and that loss to follow up is one of the eminent barriers to improving cure in TB patients 

**Author response**: We have revised the first sentence of the abstract as follows:

“Adherence to and completion of tuberculosis (TB) treatment remain problematic in many high burden countries.”

And the first sentence of the Introduction as follows:

“Ensuring that patients complete treatment for tuberculosis (TB), the leading infectious cause of death worldwide, remains a key challenge to achieving cure in many high burden countries.”

page 6, last sentence: it would be critical to add some details from the article referenced about the extent of local DOT implementation around the time that the trial was conducted. The reader needs to understand how much the SOC in Uganda matches what should be happening on paper

**Author response**: We added some additional detail in the Methods section where what occurred during the control period is described. Full details are provided in our previous paper evaluating TB treatment supervision at the trial health centers.

“In practice, there is substantial variation in how health facilities implement TB treatment supervision, including how daily dosing is observed and documented, whether treatment supporters are trained or supervised and whether patients who miss refill visits are contacted by phone or home visits [9].”

page 11, bottom lines: "However, treatment success even among patients using 99DOTS was below the 90% target specified in the END TB Strategy." isn't this partly explained by your inclusion of centres that had a treatment success <80% in previous years (the mean was only 51% treatment success as per Methods in p8)? the national reported rates of 74% in new & relapse cases in 2018 may mean that quite a few centres could reach the target values with the support of aids like 99DOTS (<https://urldefense.proofpoint.com/v2/url?u=https-3A__worldhealthorg.shinyapps.io_tb-5Fprofiles_-3F-5Finputs-5F-26entity-5Ftype-3D-2522country-2522-26lan-3D-2522EN-2522-26iso2-3D-2522UG-2522&d=DwIGaQ&c=iORugZls2LlYyCAZRB3XLg&r=0RRQgw3mvhK-sjDDiZUV3ufMG1bi_0Fmq61FgW0dg_I&m=-RDT25CPzQJ2lUvCnINqKhpv2k6oYkuYlbqbwwMm3Bk&s=0VlBE7jB7tBTJDysqr-fy78VHSqK1RyqR7Gomc3Exos&e=> ) ? can you comment some more in the Discussion about this?

**Author response**: This sentence has been removed from the revised Discussion.

page 12, para 2: here or in the last para on this page can you add one or two sentences to explain why the stepped-wedge design is expected to provide a better approach to the study of DAT when compared to other more standard methods? if you believe there are downsides please add a note in the limitations para further down. Was there risk of "contamination" between participants in the intervention and control groups when they returned to the clinics to collect medication during follow up in centres randomised to the intervention in the first months?

**Author response**: Thank you for this comment. We added an explanation of the rationale for the study design to the introduction: “The cluster-randomized design allowed us to study the 99DOTS-based intervention as it would be used in routine care. The stepped-wedge design increased acceptability among the participating health facilities and feasibility of training sites on the intervention.”

The rationale for the design is further articulated in our published protocol paper: “We chose the stepped-wedge trial design to maximise equity and acceptability (all sites will receive the intervention), minimise logistical constraints associated with introducing the intervention simultaneously at a large number of sites, account for secular outcome trends and allow for iterative learning and changes in intervention roll-out as would occur during eventual scale-up.”

The risk of contamination (control patients receiving the intervention) was low. Only 35/1,022 patients who initiated treatment during the control period were later enrolled on 99DOTS and thus excluded from the PP population. This has been clarified in Figure 1.

Page 12, midway: "Our trial extends upon these prior findings and lends further support to the increasing calls to abandon DOT and make the TB treatment experience more patient-centered" can you temper this commens with the relative disadvantages of 99DOTS as an approach when compared with direct physical interaction with a healthcare worker? in your opinion, and based on the findings, which patient risk factors and at which stage in their treatment would you think this DAT could be particularly helpful (e.g. would you posit that the improved effect in HIV-positive and in men has application beyond your study population)? do you anticipate an intervention model whereby all TB patients get 100% 99DOTS as the primary standard of care?

**Author response**: The reviewer raises excellent questions. We agree due to the limitations of the per protocol analysis it is not reasonable to conclude based on the study that all patients should receive 99DOTS. However, as was done in the trial, we believe 99DOTS should be available as an option that health workers and patients can choose. The per protocol analysis we believe indicates 99DOTS is an appropriate choice for many patients that avoids some of the inconvenience associated with DOT. In addition, since the p-value for the interaction terms were not significant, we cannot definitively conclude that 99DOTS is associated with improved effect in HIV-positive patients and men. We are currently analyzing survey and interview data related to advantages/disadvantages of 99DOTS. For now, we have highlighted other studies of implementation challenges at the end of the second paragraph of the Discussion:

“Previous observational studies of 99DOTS in India have reported variable acceptance by patients [17], sub-optimal accuracy for measuring adherence [18] and worsening of treatment outcomes following its implementation [19].”

page 13, conclusion para: do you think that the adaptations explained in supplementary figure 2 contributed to the effects observed and do you advise similar customization in the future implementation of the technology?

**Author response:** We thank the reviewer for this comment. We are currently analyzing adherence data and other data (quantitative and qualitative) around patient and health worker engagement with 99DOTS. We plan to publish this work separately.

***** Reviewer #3:** 

This study reported a stepped wedge clustered randomized trial aiming to evaluate the effectiveness of 99DOTS in improving treatment outcome among drug susceptible tuberculosis (TB) patients among 18 health facilities in rural Uganda. This is an important study to fill the gap of the lack of evidence of applying the new digital technologies in improving TB treatment adherence in low and middle income countries. The study reported a non-significant result on the primary outcome, i.e., treatment success rate, between patients under 99DOTS intervention and those in routine care based on the intention to treat (ITT) analysis, but significant improvements in the intervention group compared with control regarding primary, secondary outcomes and in sub-group analyses. The manuscript was well written and clearly presented, but needs substantial work to address the following methodological concerns regarding patient inclusion, analyses and discussion.

1.    Per protocol analysis was not mentioned in the protocol. This must be an ad-hoc analysis and have to be stated clearly the rationale of the PP analysis, and how the criteria of per protocol analysis was established.

**Author response:** We apologize this was not clear. The trial protocol was finalized prior to the start of the study and was not updated subsequently. The analysis of the trial was guided by the statistical analysis plan (SAP). The SAP was developed before completion of the trial and prior to beginning data analysis. The per protocol analysis was pre-specified and detailed in the SAP. We neglected to include the SAP in the original submission, but it is now included as “S1 Statistical Analysis Plan.” We have added the following sentence to the end of the study design and participants section of the Methods:

“The trial protocol has been published previously [8], and copies of the trial protocol, statistical analysis plan, and CONSORT checklist are available as supporting information (S1 Trial Protocol, S1 Statistical Analysis Plan, S1 CONSORT Checklist).”

In addition, we have clarified the criteria and rationale for the per protocol analysis in the Statistical analysis sub-section of the Methods:

“The ITT analysis included all eligible patients initiated on TB treatment during the control and intervention periods and thus aims to provide an unbiased estimate of the effect of the 99DOTS-based intervention in the study population overall. The PP analysis excluded patients who 1) initiated TB treatment while their health facility was in the control period but were later enrolled on 99DOTS at any point during treatment or 2) initiated TB treatment while their health facility was in the intervention period but were not enrolled on 99DOTS during the first month of treatment. The PP analysis thus aims to estimate the effect of actually receiving the assigned intervention.”

2.    There are other alterations among the secondary outcomes, comparing what was reported on page 17 of the protocol. In the manuscript, the nominator of one of the secondary outcomes, the proportion of persistence, has been changed from patients completed at least 60 doses of treatment to patients who completed the intensive phases. There were no reports on the proportion converted as in the protocol.

**Author response:** The proportion who converted smear results was removed as a secondary outcome after the trial began. The trial registration was updated to reflect this change on July 11^th^, 2019 (<https://pactr.samrc.ac.za/TrialDisplay.aspx?TrialID=3567>). There were two reasons for this change. First, many trial sites had switched over from using smear microscopy to using Xpert MTB/RIF as the primary method for TB diagnosis. Second, most trial sites did not routinely perform two-month smear examination. Thus, smear conversion data would not be available for the vast majority of patients.

Regarding persistence, the data was analyzed as specified in the protocol and documented in the Statistical Analysis Plan: “Number treated and documented as having completed at least 60 doses of treatment without being lost to follow up. These 60 doses do not need to be consecutive. Data will be obtained from the treatment register. If a patient came to pick up their Month 3 continuation phase drugs, we will assume that they completed the first 60 doses.” 60 days was always intended to be an operational definition for the intensive phase of treatment, which is 2 months for drug susceptible TB.

We clarified both points as follows:

“The primary outcome was the proportion of patients with treatment success, defined as having a treatment outcome of cured or treatment completed recorded in the TB treatment register. Pre-specified secondary outcomes included the proportion of patients completing the intensive phase of TB treatment (defined as completing 60 doses), the proportion of patients not lost to follow-up, and the proportion converted (proportion of bacteriologically-positive patients who are smear-negative at 2 months). Conversion was removed as a secondary outcome after the trial began, as the data were not available; many trial sites had switched from smear microscopy to Xpert MTB/RIF as the primary method for TB diagnosis, and most trial sites did not routinely perform two-month smear examination.”

3.    What are the adherence rates of patients on 99DOTS program? Did non-adherence happen not only in the beginning but also in other periods during the treatment, e.g., when patients become better by the end of the treatment? Within the PP analysis, why the study excluded patients who did not enrol on 99DOTS within the first month, but did include patients who stopped using or dropped out 99DOTS in subsequent months?

**Author response:** Thank you for raising these important issues. Regarding adherence, we are conducting a detailed analysis to be published separately, as we believe it deserves its own consideration. Below is a summary of the adherence data for trial patients enrolled on 99DOTS and included in the per protocol analysis:

|  | **All patients, n(%)** |
| --- | --- |
| Total number patients enrolled on 99DOTS | 463 |
| Total expected doses | 73,065 |
| Total doses due to direct calls by patients | 41,038 (56.2%) |
| Total doses recorded as taken (Direct calls and Manual doses) | 69,156 (94.6%) |
| Number of Patients with recorded doses taken > 50% | 446 (96.3%) |
| Number of Patients with recorded doses taken > 80% | 429 (92.75) |
| Number of Patients with recorded doses taken > 90% | 400 (86.4%) |
| Number of Patients with recorded doses taken 100% | 247 (53.3%) |

While overall adherence was high, particularly in the context of routine care, we are still in the process of analyzing dosing patterns and the pattern of engagement with making calls to report dosing (as opposed to doses being confirmed manually by health workers).

Regarding why we excluded patients who were not enrolled on 99DOTS within the first month of treatment, this was debated extensively when formulating the statistical analysis plan. Ultimately, we felt excluding these patients was the more conservative approach. Our thought process was that including patients enrolled on 99DOTS later into treatment could bias our results in favor of the intervention as these patients may be ***less*** likely to drop out of treatment (they had already “survived” to that point). As shown in the histogram below, only 41 of 504 (8.9%) patients enrolled on 99DOTS during the intervention period were enrolled after the first month of treatment. We repeated the per protocol analysis including these 41 patients and the odds ratio favoring the intervention is more extreme (3.55 v. 2.89), since most of them completed treatment (39/41).

Similarly, our thought process was that excluding patients who stopped using 99DOTS after initially being enrolled on 99DOTS could bias results in favor of the intervention as these patients may be ***more*** likely to drop-out of treatment. Of note, other than patients who transferred to another facility to complete treatment (such patients were excluded from both arms), only three patients enrolled on 99DOTS during the first month of treatment were noted to have discontinued 99DOTS prior to completing treatment.

4.    On Page 7 of the manuscript, the authors stated that patients who started TB treatment during the buffer period, i.e., the first month of switching from control to intervention, were excluded from primary analysis. However, in S1 Figure, the flow chart, the authors did not state the number of patients excluded for this reason. This should appear in the same line as the 566 transferred-out patients.

Table S4 indicated that patients who initiated their treatment during the buffer period were not excluded from analysis (probably included for the control period), because the total number of patients, if including those initiating treatment during the buffer period, are the same (n=2224) as before. This seems to contradict the methods section.

If these patients were included in the control period, this will dilute the difference between the two groups.

**Author response:** Thank you for pointing this out. We have clarified in S1 Figure that the 311 patients who initiated treatment during the buffer period were excluded from the study population. We have also revised Figure 1 to better reflect this.

Regarding the S4 Table (now S2 Table), it presents the results of the pre-specified sensitivity analysis including the buffer period. The Methods section describes this analysis: “A pre-specified sensitivity analysis was conducted in which patients initiating TB treatment during the buffer period were assigned to the control period if they initiated treatment on or before the first day of 99DOTS training and to the intervention period if they initiated treatment after the first day of 99DOTS training.”

We have clarified in the results: “In a pre-specified sensitivity analysis, the 311 patients from the buffer period were included in the control population if they initiated treatment on or before the date of their health facility’s 99DOTS training (n=118) and in the intervention period if they initiated treatment after the date of their health facility’s 99DOTS training (n=193).” The total N when patients initiating treatment during the buffer period are included is 2,224, as indicated in S4 Table (now S2 Table). We have also clarified the title of S4 Table (now S2 Table) to: “Pre-specified sensitivity analysis of the effectiveness of the 99DOTS-based intervention when including the buffer period.”

5.    There have been a large number of patients who did not enrol in 99DOTS program in the first month (47%, 428/ 891). What were reasons the authors may identify from the process evaluation? These patients apparently had much worse outcomes compared with the rest of their peers in the intervention group/period. This is important message that needs to be discussed.

**Author response:** In the fourth paragraph of the Results section, we present the reasons for non-enrollment on 99DOTS: “Reasons for non-enrollment on 99DOTS included lack of regular access to a phone (n=277, 64.7%), patient preference (n=33, 7.7%), death/loss to follow-up before enrollment (n=28, 6.5%), being deemed too ill or elderly by health facility staff (n=18, 4.2%), work- or school-related reasons (n=5, 1.2%), and unknown (n=67, 15.7%).”

We are conducting interviews with patients and providers to further understand barriers to enrollment. We have also clarified in the Results and in Figure 1 that the 428 patients not enrolled on 99DOTS during the first month includes those who did not enroll in 99DOTS at all and those who enrolled later in treatment:

“The remaining 428 were either never enrolled on 99DOTS (n=387) or enrolled later on in treatment (n=41).”

6.    In Table S5, there is a large number of patients who did not register a telephone number in the records. Were these patients provided with a cell phone? On page 19, the authors reported that 99DOTS was not used for a substantial proportion of the patients. What is the rate of patients using the 99DOTS? There are a number of related outcomes in the category of Implementation outcomes. This needs to be either reported or quoted from your other publications. This a crucial to understand the effectiveness of 99DOTS. It seems that the author indicated that the acceptability/ usability of 99DOTS were low. So the program is only effective among patients who use it.

**Author response:** Patients were not provided with a cell phone; those who lacked regular access to a cell phone were not able to enroll on 99DOTS. As noted in our response to comment 5, we have clarified the number of patients not enrolled on 99DOTS vs. those who were enrolled later in treatment. This paper focused on the reach and effectiveness outcomes of the trial. We are preparing a separate mixed methods paper focused on the implementation outcomes.

I would suggest the authors would focus on the possible reasons regarding the difference between the ITT and PP analyses in the discussion section.

**Author response:** We agree with this suggestion and have added the following text to the new third paragraph of the Discussion:

“Due to the pragmatic nature of our trial, health workers and patients made all decisions about the use of 99DOTS during the intervention period, and only about half of all eligible patients were enrolled on 99DOTS. The substantially better treatment outcomes among these patients in the per protocol analysis should not be interpreted as improving outcomes in the overall population, primarily due to the possibility of selection bias. Patients enrolled on 99DOTS likely represent a non-random sample of patients initiating treatment with higher interest in engaging in care and therefore higher likelihood of completing treatment even without 99DOTS. Although our post-hoc analysis excluding patients without listed phone numbers showed similar findings to the per protocol analysis, it may not adequately control for this bias – the reason for non-enrollment on 99DOTS was unrelated to phone access for approximately one-third of patients in the intervention period.”

***** Reviewer #4:** 

This is a well-conducted stepped-wedge cluster RCT on digital adherence technology for tuberculosis treatment supervision. The study design, dataset, statististical methods and analyses, and presentation (tables and figures) and interpretation of the results are mostly adequate and of a good standard. However, there are still some issues needing attention.

1) Sample size calculation. With the information on Page 10, one is unable to reproduce the sample size and power. More details of the sample size calculation (parameters) are needed. How many clusters in the calculation? is it 18 or 6? How many steps? what's the cluster size? what's justificaiton of the assumption of 10% increase in the treatment success proportion? Where is the ICC of 0.001 from and any justification? Stepped-wedge design is complex so that sample size calculation needs to be very clear and detailed.

**Author response:** We have clarified that the sample size calculation used 18 clusters and 6 steps:

“With 18 clusters (health facilities), an anticipated harmonic mean of 15 eligible patients per month at each health facility, 6 steps (three health facilities randomized to the intervention each month), and a type 1 error (alpha) of 0.05, the trial had 89% power to detect a 10% increase in the treatment success proportion, comparing the intervention relative to the control period [8]. The power calculation assumed a pre-implementation treatment success rate of 51% and intraclass correlation coefficient of 0.001 (both determined using 2017 Uganda NTLP data from participating health facilities).”

The cluster size is indicated by the harmonic mean number of eligible patients initiated on treatment each month. As stated in the Methods, the ICC was determined using 2017 Uganda NTLP data from participating health facilities. A full description of the sample size calculation is available in the published protocol paper (Crowder R et al. Study protocol and implementation details for a pragmatic, stepped-wedge cluster randomised trial of a digital adherence technology to facilitate tuberculosis treatment completion. BMJ Open. 2020;10(11):e039895. doi:10.1136/bmjopen-2020-039895). We defer to the editor whether these details should also be included in this manuscript.

2) The ITT vs PP analysis is the key of the paper. However, the definiton of the PP analysis "The PP analysis excluded patients who initiated treatment during the control period but were nonetheless enrolled on 99DOTS prior to treatment completion" is a bit difficult to understand. Can authors please explain what exactly this means?

**Author response:** We have clarified in the Statistical analysis section:

“The ITT analysis included all eligible patients initiated on TB treatment during the control and intervention periods and thus aims to provide an unbiased estimate of the effect of the 99DOTS-based intervention in the study population overall. The PP analysis excluded patients who 1) initiated TB treatment while their health center was in the control period but were later enrolled on 99DOTS at any point during treatment or 2) initiated TB treatment while their health center was in the intervention period but were not enrolled on 99DOTS during the first month of treatment. Although limited by the potential for selection bias, the PP analysis aims to estimate the effect of actually receiving the assigned intervention.”

***** Reviewer #5:** 

Thank you for the opportunity to review this interesting submission.  I have a number of comments and suggestions, but I do believe that this paper presents information important to the discipline (improving TB treatment/health outcomes through the use of innovative, patient-centered technologies) and is meritorious of publication.  The paper correctly points out that, while there are many studies and publications addressing the use of digital adherence technologies to improve medication adherence, there are relatively fewer studies and publications (i) involving TB, (ii) conducted in highly pragmatic settings with a view toward generalizability with respect to other high burden regions, and, most importantly, (iii) focused on treatment/health outcomes as a primary endpoint.  Especially given the other aspects of this study that have been published (user-centered design aspects) and are indicated as to be published (cost-effectiveness aspects), this study/paper represents
the centerpiece of what is probably the most comprehensive evaluation to date of the use of digital adherence technologies as an enabler/enhancer of TB care.  Again, I think it merits publication.

**Author response:** We thank the reviewer for their overall positive assessment of our manuscript. Please find below responses and changes based on specific comments.

My specific comments and questions to the authors are set forth below:

1. My most important question/comment related to the primary end point and the distinction drawn between ITT and PP populations.  I understand the distinction and the figures did an OK (not great job) of showing the differences in these patient populations.

**Author response:** We thank the reviewer for raising this. We have revised Figure 1 to better describe the study including the differences between the ITT and PP population. We have moved Figure 2 to the Supplement (now S2 Figure) as it seemed redundant after the revisions to Figure 1.

However, given the extremely important differences in outcomes (no significant effect in ITT and quite significant effect in PP), I think that the authors should be clearer about which is the more relevant group/more significant finding and why.  It would seem that one could argue that the PP population/finding is more important in that more patients in this group seem to have received the actual intervention in the intervention arm.  However, the PP group also excludes a number of "typical" patients so maybe it is an unrepresentative population.  I would like to see the ITT/PP distinction and significance made clearer if possible.  The authors stater on page 28 that "in conclusion, our trial provides evidence from a
randomized trial that 99DOTS-based treatment supervision is effective for adults with pulmonary TB who are enrolled on the platform."  Why isn't that the true bottom line here, rather than looking to an ITT population where the results seem more affected by "reach" than "efficacy" of the intervention?

**Author response:** We thank the reviewer for raising this issue and agree this was not clear.

We have included the following revised text to better clarify the distinction between the ITT and PP populations:

“Primary and secondary outcomes were analyzed according to intention-to-treat (ITT) and per protocol (PP) principles. The ITT analysis included all eligible patients initiated on TB treatment during the control and intervention periods and thus aims to provide an unbiased estimate of the effect of the 99DOTS-based intervention in the study population overall. The PP analysis excluded patients who 1) initiated TB treatment while their health center was in the control period but were later enrolled on 99DOTS at any point during treatment or 2) initiated TB treatment while their health center was in the intervention period but were not enrolled on 99DOTS during the first month of treatment. Although limited by the potential for selection bias, the PP analysis aims to estimate the effect of actually receiving the assigned intervention.”

Also, based on this comment and comments from other reviewers, we have substantially revised the discussion. We agree with reviewers overall that the ITT results should be more emphasized and the concerns about bias with the per protocol analysis better articulated. We hope the revised Discussion (as summarized in our response to Reviewer 1, Major critiques 1 and 2) better highlights that 99DOTS did not improve overall treatment outcomes and based on this trial cannot be considered as a replacement for DOT. However, we do think based on the per protocol findings, 99DOTS should be offered as an alternative to DOT and that patients (and health workers) should be able to choose between the two as was essentially done in the trial.

2. Related to point #1 above, I found the abstract to be underwhelming (compared to the well-stated conclusions and insights on pages 19-20).  I wonder if some improvements might be possible/in order.

**Author response:** We have revised the abstract conclusion based on this comment and to be consistent with changes in the Discussion.

3. On page 6 of the paper, there is a reference to $4-6/patient at scale.  Have the authors validated that pricing in Uganda or is this based on 99DOTS or other information?  I am not sure the parenthetical adds value (maybe low-cost alone is sufficient) and may be provocative if not substantiated as real in the Uganda context.

**Author response:** We agree this does not add value here and have removed “$4-6/patient at scale.” Costing of the 99DOTS-based intervention we used in Uganda will be presented in detail in a separate publication.

4. I very much like the secondary end points related to persistence through intensive phase and also loss to follow up.  Given the potential impact of these aspects on both transmission and recurrence, I applaud the study design and inclusion of these additional end points.

**Author response:** Thank you for these comments.

5. I wonder if it is worth noting in the discussion and conclusions that Uganda seems to have primarily community as opposed to facility-based DOT.  That is noted earlier in the paper, but it might be worth noting that the conclusions supporting use of 99DOTS vis a vis DOT are in that (community-based rather than facility-based) context.

**Author response:** We appreciate this comment but prefer to generically refer to DOT rather than specify community-based DOT – as facility-based DOT is not feasible to provide to all patients in such resource-limited settings where daily transport to a facility is often unfeasible for patients. Previous studies have not shown a difference in outcomes with any form of DOT, nor significant differences between community- and facility-based DOT (<https://www.cochranelibrary.com/cdsr/doi/10.1002/14651858.CD003343.pub4/full>). Ultimately, we are willing to make this change if strongly desired by the reviewer, but we worry that it could have the unintended consequence of suggesting that facility-based DOT should be more broadly implemented – in a population for whom facility-based DOT is not a patient-centered option.

6. I thought the description of the 99DOTS intervention was clear.  I wonder if it might be worth adjusting language slightly to highlight that the "hidden number" is a feature designed to increase confidence that the patient has "engaged" with his/her medication and therefore the dosing signal is more accurate.  Not a major point.

**Author response:** We have revised slightly as follows:

“99DOTS (Everwell Health Solutions, India) is an alternative low-cost DAT that involves patients calling toll-free phone numbers that are ordered in an unpredictable pattern and hidden underneath pills in blister packs [5,6]. The phone numbers are revealed only when patients remove scheduled medication doses from the blister pack, enabling patients to make toll-free calls to self-report medication dosing. Health facility staff can access adherence data for individual patients in real-time through a web dashboard and mobile phone application.”

7. I did have to get out a calculator to determine how the populations in the ITT and PP groups were calculated.  I wonder if a figure showing how these groups were derived and the various exclusions applied in getting to the PP group might be helpful.

**Author response:** We thank the reviewer for raising this. We have revised Figure 1, as noted in our response to Reviewer 5’s first comment. The total N in each period is equal to the sum of all patients in the month-specific boxes from that period. The ITT population included 100% of these patients, while the PP population had exclusions noted in the updated Figure 1: 1) 35 patients excluded from the control period because they were later enrolled on 99DOTS, 2) 387 patients excluded from the intervention period because they were never enrolled on 99DOTS, and 3) 41 patients excluded from the intervention period because they were enrolled on 99DOTS after the first month of treatment.

8. Again, just to reinforce the point -- the paragraph on page 15 is a very strong statement of the effectiveness of 99DOTS (in treatment outcomes, in persistence through IP phase, and in LTFU -- 3X more likely to complete treatment) for the PP group.  If that is really the more relevant group, these findings are really important.  But is it -- or is the ITT group the more relevant.  Unclear.

**Author response:** As noted in our response to Comment 1, we have tried to clarify this issue in the revised Discussion.

9. The Kenya study referenced on page 18 had, I believe, a health outcomes impact/end point.  Are the authors comfortable with the characterization on page 18?

**Author response:** We have removed the following sentence from the revised Discussion:

"To our knowledge, these data represent the first to show the impact of a low-cost DAT on TB treatment outcomes."

10. On page 19, the authors state: " Even though overall treatment outcomes did not improve . . . " and then goes on to talk about other, ancillary benefits of 99DOTS (empowerment, etc.)  Is that statement correct and/or appropriate?  Elsewhere in the paper the authors make it clear that 99DOTS did in fact improve outcomes for patients enrolled thereon.  Should this statement on 19 be revised?  IF the ITT result was more about reach than effectiveness (do the data give any insights on this?), then that is not clear -- especially in statements like the one referenced.  

**Author response:** We have revised the Discussion substantially, taking this comment and those from other reviewers into account. We hope the revised Discussion is clearer in terms of how we view the ITT vs. per protocol analyses.

I hope these comments are helpful, and thanks to the authors for their efforts on behalf of TB patients globally.

**Author response:** The comments were very helpful, and we thank the reviewer for taking the time to provide a detailed and thorough review to help us improve the manuscript.
