# Supplementary material for: Digital adherence technology for tuberculosis treatment supervision: A stepped-wedge cluster-randomized trial in Uganda
Source: PLoS Med. 2021 May 6;18(5):e1003628. doi: 10.1371/journal.pmed.1003628 (PMC8136841; doi:10.1371/journal.pmed.1003628)
Supplement: Attachment — Submitted filename: PLOS Med Response to Reviewers_2021.4.8.docx [file pmed.1003628.s012.docx]

**Response to reviewers**

**PLOS Medicine Manuscript Number PMEDICINE-D-20-06186R2**

Dear Editorial Board,

Thank you for the opportunity to submit a revised draft of our manuscript entitled “Digital adherence technology for tuberculosis treatment supervision: a stepped-wedge cluster randomized trial in Uganda.” We appreciate the editor’s and reviewers’ additional comments. Please see below, in blue, our responses to the comments.

**Requests from Editors:**

Please quote the study setting (Uganda) in your title.

**Author Response:** We have added the setting to the title: “Digital adherence technology for tuberculosis treatment supervision: a stepped-wedge cluster randomized trial in Uganda”

Please quote aggregate demographic details for participant groups in the abstract.

**Author Response:** We have added the following sentence to the abstract: “Of 1,913 eligible patients at the 18 health facilities (1,022 and 891 during the control and intervention periods, respectively), 38.0% were women, mean (SD) age was 39.4 (14.4) years, 46.8% were HIV-infected and most (91.4%) had newly-diagnosed TB.”

We ask you to de-emphasize the per-protocol findings in your abstract and main text. In the abstract, we suggest quoting the ITT findings, followed immediately by a summary of the subgroup findings by sex and HIV status (noting that these are non-significant), with the per-protocol findings summarized briefly thereafter.

**Author Response:** We have revised the results in the abstract to read:

“In the ITT analysis, the odds of treatment success were similar in the intervention and control periods (adjusted odds ratio [aOR] 1.04, 95% CI 0.68-1.58, p=0.87). The odds of treatment success did not increase in the intervention period for either men (aOR 1.24, 95% CI 0.73-2.10) or women (aOR 0.67, 95% CI 0.35-1.29), and for either patients with HIV infection (aOR1.51, 95% CI 0.81-2.85) or without HIV infection (aOR 0.78 95% CI 0.46-1.32). In the per protocol analysis, the 99DOTS-based intervention increased the odds of treatment success (aOR 2.89, 95% CI 1.57-5.33, p=0.001). The odds of completing the intensive phase of treatment and the odds of not being lost to follow-up were similarly improved in PP but not ITT analyses.”

Please avoid claims such as "the first" (e.g., in your author summary) and where needed add "to our knowledge" or similar.

**Author Response:** We have removed such claims throughout.

Please trim the author summary so that each subsection contains 3-4 short points.

**Author Response:** We have shortened and/or removed bullets as requested.

Similarly, in the main text please present the subgroup analyses before the per-protocol findings.

**Author Response:** We have moved the ITT sub-group analysis before the per-protocol findings as requested.

Throughout the text, please use the style "the 4 randomized trials" (though numbers should be spelt out at the start of sentences).

**Author Response:** We have replaced spelled-out numbers with numerical digits throughout the text.

**Comments from Reviewers:**

***** Reviewer #1:**

My only final piece of feedback is that, given that the authors suggest that 99DOTS may be a viable alternative to DOT for some patients in this setting, they should provide a better description of what the "control" condition of DOT was that they were actually comparing against. "Community DOT" can mean many different things, and the authors note that there is considerable variability in implementation of community DOT. With that said, it would still be critical for readers to know:

(1) In general (acknowledging that there may be variability) who most commonly serve as treatment supporters? Are they patients' family members—such that it is relatively convenient/easy for patients to be observed? Are they other people living in the community? Are they local healthcare workers?

(2) Are treatment supporters paid or compensated by the government?

(3) Are there any other costs of DOT in this context that would not also be there under 99DOTS-based monitoring? (Authors do not need to provide the exact costs but just very briefly—in a few words—note other aspects of care where costs may be incurred that might be different with DOT as compared to 99DOTS).

**Author Response:** We have added additional detail as requested and more clearly indicated that a previous publication has described treatment supervision practices at the 18 health facilities:

“During the control period, health facilities continued providing TB treatment supervision in the same manner as before the trial. The community-based DOT model used at the 18 health facilities has been described previously, including the substantial variation in how TB treatment supervision is implemented across the facilities [9]. All facilities request patients to take their TB medicines under direct observation by a treatment supporter. The treatment supporter is most commonly a family member who receives no training or compensation. All health facilities report assessing adherence (most commonly by patient self-report) at drug refill visits scheduled every 2 weeks during the intensive phase and every 4 weeks during the continuation phase. Patients who miss refill visits are not followed-up at the majority of health facilities.”

Two or three sentences in the Methods better describing what community DOT means in this context—even while recognizing that the model may be variable—is important for readers to be able to assess the authors' final claim that 99DOTS may help some patients avoid the "inconvenience and additional costs of directly observed therapy."

The authors don't provide enough basic information for the reader to assess whether this could potentially be true. For example, if "treatment supporters" are simply unpaid family members, this would essentially be free for the government and therefore cheaper than 99DOTS, which involves extensive printing of paper envelopes, personnel time to pack medication blister packs in these envelopes and review the dashboard, tech support costs, lost time for patients to call phone numbers on a daily basis, and other costs. On the other hand, if treatment supporters are usually paid non-family members then it would be clear that 99DOTS may potentially be cheaper to the government and more convenient for some patients, as the authors claim.

This clarity would be very helpful to readers. A major problem with the TB digital adherence technologies (DATs) literature is that the "control" condition can be so variable across contexts and is often poorly defined in papers that are trying to assess whether these improve outcomes.

**Author Response:** We have added some additional detail as described in the previous response. We do not make any claims that 99DOTS is cost-saving for the health system. However, it may help reduce workload and increase efficiency of health workers by helping them focus on non-adherent patients and making adherence data readily available. We are analyzing data from time and motion studies and will report health system costs in detail in a future publication. From a patient perspective, the need to be observed taking treatment has been described by many TB survivors as inconvenient and stigmatizing, regardless of whether the treatment supporter is a family member or health worker. With respect to costs, we agree in this setting costs are less of an issue for most patients (but not all) in that a family member is the one observing treatment. But cost savings are a bigger issue in settings that do facility-based DOT and, to a lesser extent, in settings that require a patient to visit a local (non-family member) treatment supporter. Thus, we believe our claim about reducing inconvenience is valid for most patients (regardless of setting) and the part about reducing costs is valid for some patients in Uganda but many patients in other settings.

***** Reviewers #3 and #4: No additional comments to address.**
